# A comprehensive pan-cancer analysis of necroptosis molecules in four gynecologic cancers

**DOI:** 10.1186/s12885-022-10166-6

**Published:** 2022-11-10

**Authors:** Jianfeng Zheng, Xintong Cai, Yu Zhang, Huihui Wang, Li Liu, Fengling Tang, Linying Liu, Yang Sun

**Affiliations:** 1 Department of Gynecology, Clinical Oncology School of Fujian Medical University, Fujian Cancer Hospital, No.420, Fuma Road, Jin ‘an District, Fuzhou City, 350014 Fujian Province People’s Republic of China; 2 Department of Preventive Medicine, School of Public Health, Fujian Medical University, Fuzhou, 350122 China

**Keywords:** Gynecologic cancer, Necroptosis, Prognosis, Immunity, Immunotherapy

## Abstract

**Background:**

In recent years, it has been proved that necroptosis plays an important role in the occurrence, development, invasion, metastasis and drug resistance of malignant tumors. Hence, further evaluation and targeting of necroptosis may be of clinical benefit for gynecologic cancers (GCs).

**Methods:**

To compare consistency and difference, we explored the expression pattern and prognostic value of necroptosis-related genes (NRGs) in pan-GC analysis through Linear regression and Empirical Bayesian, Univariate Cox analysis, and public databases from TCGA and Genotype-Tissue Expression (GTEx), including CESC, OV, UCEC, and UCS. We explored the copy number variation (CNV), methylation level and enrichment pathways of NRGs in the four GCs. Based on LASSO Cox regression analysis or principal component analysis, we established the prognostic NRG-signature or necroptosis-score for the four GCs. In addition, we predicted and compared functional pathways, tumor mutational burden (TMB), somatic mutation features, immunity status, immunotherapy, chemotherapeutic drug sensitivity of the NRG-signature based on NRGs. We also examined the expression level of several NRGs in OV samples that we collected using Quantitative Real-time PCR.

**Results:**

We confirmed the presence of NRGs in expression, prognosis, CNV, and methylation for four GCs, thus comparing the consistency and difference among the four GCs. The prognosis and independent prognostic value of the risk signatures based on NRGs were determined. Through the results of subclass mapping, we found that GC patients with lower risk score may be more sensitive to PDL1 response and more sensitive to immune checkpoint blockade therapy. Drug susceptibility analysis showed that, 51, 45, 64, and 29 drugs with differences between risk groups were yielded in CESC, OV, UCEC, and UCS respectively. For OV, the expression differences of several NRGs in the tissues we collected were similar to that in TCGA.

**Conclusion:**

Our comprehensive analysis of NRGs and NRG-signature demonstrated their similarity and difference, as well as their potential roles in prognosis and could guide therapeutic strategies, thus improving the outcome of GC patients.

**Supplementary Information:**

The online version contains supplementary material available at 10.1186/s12885-022-10166-6.

## Introduction

Gynecological cancers (GCs) are the main types of female cancers, among which uterine and ovarian cancers are the most common [1]. Despite the high response rate to chemotherapy, most ovarian cancer patients develop resistance to first-line chemotherapy drugs and these patients have a poor prognosis [2], hence, the mortality rate of ovarian cancer is the highest among gynecological tumors [3]. Cervical cancer is the second most common malignancy in women, and although the development of early diagnosis methods has improved the rate of early detection of cervical cancer, the prognosis for patients in advanced stages remains poor [4]. The high incidence and mortality rate of GCs have seriously jeopardized women’s life, health and quality of life [5]. Therefore, new therapeutic strategies are urgently needed to improve the prognosis of patients with GCs.

Orderly cell proliferation and death is an important condition to maintain the homeostasis of intracellular environment. Tumors may arise when cells in the body overproliferate in a disorder that cannot be killed by normal pathways of death. Uncontrolled proliferation and resistance to cell death are two major markers of malignant tumors [6]. Apoptosis, which was first proposed by Kerr JF et al. in 1972, is the earliest known programmed cell death phenomenon [7]. In 2005, Degterev A et al. found that the form of cell necrosis caused by cell membrane rupture caused by RIPK3 activation of MLKL also has a programmed regulation phenomenon, which is called necroptosis [8]. Necroptosis has similar cell morphology to necrosis (early destruction of envelope integrity, cell volume and intracellular organelle swelling), but differs from necrotic apoptosis in that it is a regulated caspase-independent programmed necrosis [9]. With the development of basic research, it is found that necroptosis is not only related to the mechanism of inflammatory pathology [10], but also closely related to the occurrence, development and drug resistance of tumors [11]. It has been proved that necroptosis and targeting necroptosis has dual effects on tumor occurrence and development [12]. Specifically, it has been shown that in pancreatic ductal adenocarcinoma (PDA), when RIPK3 knockout tumor cells undergo necroptosis, soluble cytokines released bind to receptors on inflammatory cells, triggering an immunosuppressive tumor microenvironment and promoting the progression of PDA [13]. Necroptosis induced inflammatory response may lead to metastasis of breast cancer [14]. Inducting apoptosis was proven with low efficacy because of apoptosis-resistance, hence, triggering necroptosis is a novel and effective strategy [15]. Necroptosis may also kill normal cells, lead to inflammatory reactions and promote the occurrence, development, invasion and metastasis of tumors while playing an anti-tumor effect [16]. RIPK3, a key kinase of necrotizing apoptosis, is significantly down-regulated in human colorectal cancer (CRC), and its expression has anti-inflammatory and anti-tumor effects in the intestine [17]. Hence, necroptosis may serve as a double-edged sword in cancer.

Some progress has been made in the study of necroptosis, but there are still the following problems: First, the relationship between necroptosis and other forms of cell death and its intersection is complicated, which brings difficulties to clinical research [18, 19]; Second, necroptosis lacks specific molecular markers [20]; Thirdly, the mechanism of necroptosis and its regulation are still unknown and need to be further elucidated [20]. Fourthly, most studies on necroptosis are based on in vitro experiments [21]. Therefore, the in vivo effect of necroptosis on tumor cells still needs to be explored. At present, the triggering of necroptosis has been found to be effective in the treatment of colon cancer and hematological tumors [22], but its clinical efficacy in other tumors is still controversial. The study of necroptosis may open a new field of cancer research and provide a broad prospect for the development of new anticancer drugs. Although the functions of necroptosis in tumorigenesis have been confirmed preliminarily [23–38], at present, the preliminary studies mainly focus on ovarian cancer, while the research on UCEC and UCS is still blank. Overall, little is known about necroptosis in GCs. To further explore the mechanism of necroptosis and study its role in the occurrence and progression of malignant tumors and antitumor drugs will provide clinical significance for the treatment of GCs.

In this study, we performed a comprehensive evaluation to show the landscape of necroptosis-related genes (NRGs) in GCs and to explore the underlying mechanisms, thus developing strategies for diagnosis and treatment. The gene expression heterogeneity, copy number variation (CNV), and methylation level of 76 NRGs in four GCs were analyzed. Using least absolute shrinkage and selection operator (LASSO) Cox regression analysis and principal component analysis we identified necroptosis-score and constructed the reliable NRG-related prognostic signature to predict overall survival (OS). Our data showed that the NRG-related prognostic signature was associated with immunity characteristics, immunotherapy, and chemotherapeutic drug sensitivity. In a word, the signature we developed may provide new insight into GC treatment and prognosis.

## Materials and methods

### Data collection

We obtained processed datasets (TCGA TARGET GTEx) and clinicopathological data of four GCs, including CESC (cervical squamous cell carcinoma and endocervical adenocarcinoma), OV (ovarian serous cystadenocarcinoma), UCEC (uterine corpus endometrial carcinoma), and UCS (uterine carcinosarcoma), from UCSC -Xena platform [39]. According to the gene annotation information (hg38, gencode.v23.annotation.gene.probemap) [40] in GENCODE database (httRiskscore://www.gencodegenes.org/) [40], we converted the Ensemble Gene into Gene Symbol. After filtration of low-expression genes, expression matrices of mRNA were obtained according to gene annotation information. Necroptosis-related genes (NRGs) were screened from Gene Set Enrichment Analysis (GSEA) (http://www.gsea-msigdb.org/gsea/index.jsp) and previous reports about necroptosis [41, 42]. By matching with the mRNA expression matrices, 76 NRGs were finally matched, and the mRNA expression matrices related to necroptosis of a total of 31 tumors was extracted. Perhaps due to the different sequencing depth of each tumor, individual NRGs were not annotated in individual tumors and the tumors were excluded from this study.

### Differential and prognostic analysis of NRGs

Linear regression and Empirical Bayesian provided by limma package (Version3.10.3, http://www.bioconductor.org/packages/2.9/bioc/html/limma.html) [43] were used to analyze the differential expression of NRGs for the four GCs (Tumor versus Normal), respectively, thus obtaining the corresponding P value and logFC. In addition, Benjamini & Hochberg was used for multiple test correction to obtain the corrected P value (adjusted *P*-value). We evaluated from two levels of difference multiple and significance, and the threshold of difference expression was set as follows: adjusted *P*-value < 0.05&|logFC|> 0.5. Then prognostic NRGs were screened using the Univariate Cox analysis of survival package and GSCA database. In order to determine the somatic mutations of NRGs, we generated the Mutation Annotation Format (MAF) in TCGA using the “maftools” R package [44].

### Principal component analysis for NRGs

PCA (principal component analysis) analysis was performed based on the expression values of NRGs in each sample, and this step was performed based on normal, tumor and all tissues respectively, to observe whether there was a specific necroptosis pattern among the four GCs [45]. The prognostic NRGs were selected for PCA analysis to construct the necroptosis-score [46].

### Establishment of the prognostic signature based on NRGs

The NRGs with survival prognosis were screened using the Univariate Cox analysis of survival package [47] to obtain prognostic NRGs (*P* < 0.05). The samples were randomly divided into training set, validation set and total set in the ratio of 7:3 [48–52]. The training set was used for subsequent model construction, and the validation and total sets were used for model verification. Further, we used the LASSO Cox regression model [53] of glmnet package (version 2.0–18, httRiskscore://cran.r-project.org/web/packages/glmnet/index.html) [54] to further screen the combination of prognostic NRGs and obtain the prognostic coefficient of each NRG by 20-fold cross-validation analysis. Next, according to the LASSO regression prognostic coefficient of each NRG and the expression level of mRNA in TCGA, the risk-score (RS) model was constructed as follows:

$$\mathrm{Risk Score}={\sum\upbeta }_{\mathrm{NRG}}\times {\mathrm{Exp}}_{\mathrm{NRG}}$$

Here, β_NRG_ represented the LASSO regression coefficient of model gene, and Exp_NRG_ represented the expression level of NRG in TCGA dataset. And then we took the median value of the RSs, TCGA sets were divided into high-risk group (HRG, with a RS higher than or equal to the median value of RSs) or low-risk group (LRG; RS lower than the median value of RSs). Kaplan–Meier curves of survival packages were used to assess the association between survival outcomes and the risk groups.

To determine whether the RS model based on NRGs was as an independent prognostic factor, we performed Univariate Cox regression analysis for risk groups and clinicopathological parameters. Variables with *P* < 0.05 were included in Multivariate Cox regression analysis, and variables with *P* < 0.05 were screened out to draw Normgram.

### Characteristics of functional pathways, immunity status, and anti-tumor therapy

GSVA (Gene set variation Analysis) algorithm was used to calculate the enrichment scores of each HALLMARK gene set in each sample of four GCs by using GSVA package [55]. The enrichment background was h.all.v7.4.symbols in MsigDB V7.1 database [56] and the scoring matrices were obtained. Then, limma package of R was used to analyze the difference between HRG and LRG, and the corresponding t score with P values were obtained. The greater the absolute value of t score was, the more significant the difference was considered.

The occurrence and development of tumor is also closely related to the immune microenvironment [57]. CIBERSORT was a tool for deconvolution of expression matrix of immune cell subtypes based on linear support vector regression [58, 59]. We used CIBERSORT to calculate the proportion of 22 types of immune cells based on the expression levels of all genes in GC samples, and Wilcox test was used to compare whether there were significant differences in each type of immune cells between the HRG and LRG. Stromal cells and immune cells in malignant solid tumors are believed to play an important role in tumorigenesis, development and drug resistance [60]. Further, we estimated the immune score, stromal score, ESTIMATE score, and tumor purity using ESTIMATE algorithm [60]. By feeding in the gene expression matrix, we could obtain scores for the levels of immune cells, stromal cells, and the purity of the tumor sample [60]. In general, the higher the purity of the tumor sample, the more malignant it is [60].

We predicted potential responses to ICB using the TIDE algorithm [61]. We also compared the discrepancy between the two risk groups in immunotherapy using submap [62]. The chemotherapy drugs were extracted from the GDSC database (https://www.cancerrxgene.org/) [63] and used R3.6.1 pRRophetic [64] to assess IC50 levels.

To identify somatic mutations between HRG and LRG in GC patients, the “maftools” R package was used to generate the Mutation Annotation Format from the TCGA database [44]. And the tumor mutation burden score for each patient with OC in both two groups were also calculated.

### Quantitative real-time PCR

After approval by the Ethics Committee of Fujian Cancer Hospital, we collected 30 OC tissue samples and 10 normal ovarian tissues to perform Quantitative Real-time PCR (qPCR). Total RNA from the tissue samples was extracted applying TRNzol Universal Reagent from Tiangen Biotech (Beijing, China). Following RNA extraction, we reverse transcribed total RNA using FastKing gDNA Dispelling RT SuperMix from Tiangen Biotech (Beijing, China) to obtain cDNA. In order to detect the expression level of NRGs in the signature, we conducted qPCR using SuperReal PreMix Plus from Tiangen Biotech (Beijing, China). We purchased the primers of the NRGs from Sangon Biotech (Shanghai, China) and the sequences were listed in Supplementary Table 1.

### Statistical analyses

Statistical analyses and data plotting were performed using R program or GraphPad Prism 9. Spearman’s correlation analysis test was used to analyzed the correlation relationship in data types. A threshold of 0.05 was used to deem significance from p values of statistical tests. Other special analyses have been described in the previous section.

## Results

### Expression differences and prognostic value of necroptosis-related genes

**Fig. 1:**
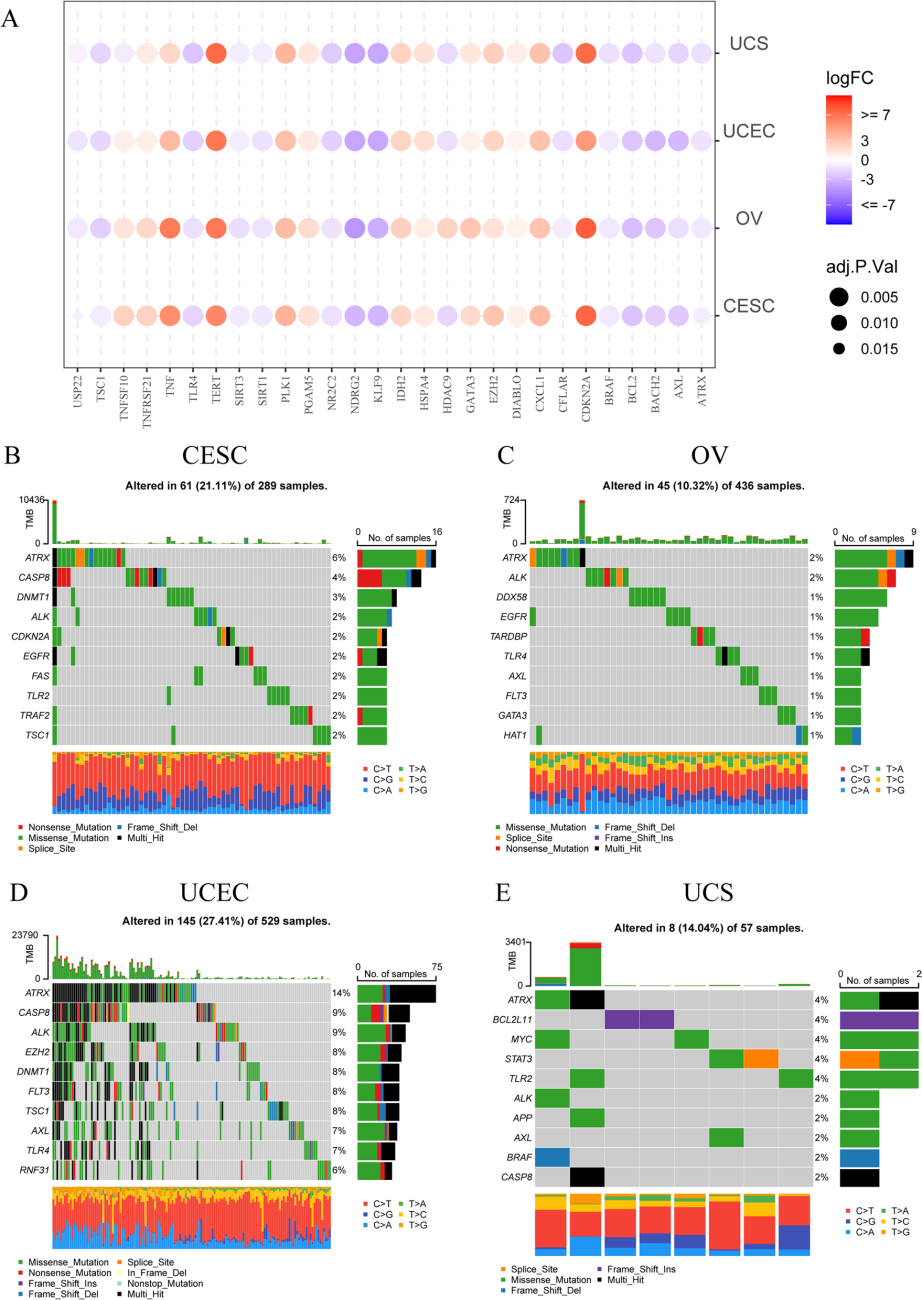
Expression variation and mutation frequency of filtered necroptosis-related genes (NRGs). **A** Expression levels of mutual differentially expressed NRGs in the four gynecological cancers (GCs). The color of the dots represents the degree of variance. Redder dots represent higher expression in cancer tissue. Bluer dots represent higher expression in normal tissue. The size of the bubbles indicates the adjusted *P*-value. Larger bubbles represent a lower adjusted *P*-value. The NRGs with adjusted *P*-value < 0.05 & |logFC|> 0.5 and NRGs that were significantly differentially expressed in all the four GCs were retained to produce the figure. **B-E** Top 10 NRGs with mutation rates in patients with CESC (**B**), OV (**C**), UCEC (**D**), and UCS (**E**). The small figure above shows the TMB, the number on the right shows the mutation frequency of each NRG, and the figure on the right shows the proportion of each variant

We explored differences in expression of 76 NRGs between normal and cancer tissues in 31 tumors. The results of the pan-cancer analysis showed that all the 76 NRGs were abnormally expressed in one or more tumors **(**Supplementary Table 2). CDKN2A was highly expressed in a total of 28 tumors, ranking first, while FASLG was only highly expressed in seven tumors (Supplementary Table 2). Comparing the expression levels of 76 NRGs in TCGA samples, several NRGs (ATRX, AXL, BACH2, BCL2, BRAF, CFLAR, KLF9, NDRG2, NR2C2, SIRT1, SIRT3, TLR4, TSC1, USP22) were found at lower expression levels in four gynecological tumors (Fig. 1A), and several NRGs (CDKN2A, CXCL1, DIABLO, EZH2, GATA3, HSPA4, IDH2, PGAM5, PLK1, TERT, TNF, and TNFRSF21) were found at higher expression levels in four gynecological tumors (Fig. 1A). Some of the differential NRGs are unique to a particular GC (Supplementary Figure S1E), such as BCL2L11 and OTULIN were unique differential NRGs of CESC, MAP3K7 was unique differential NRGs of OV, and TLR2 was unique to UCS. It was worth mentioning that RNIP3, CASP8, DDX58, FLT3, HDAC9, ID1, LEF1, RIPK3, TLR3, and TNFSF10 were found heterogeneous among the four GCs (Supplementary Figure S1E). However, in general, there was no significant difference in the number of genes with high or low expression among the four gynecological tumors. For the four GCs in the TCGA database, more than 20% of the samples had mutated NRGs and ATRX had the highest mutation frequency (Supplementary Figure S1A-D, Fig. 1B-E). Overall, UCEC (Supplementary Figure S1C) had the most mutations, while OV had the fewest (Supplementary Figure S1B). After evaluating the correlation of expression values, we found the correlations among NRGs were mostly positive in the four GCs, whether they have synergistic effects remains to be explored (Supplementary Figure S2).

**Fig. 2:**
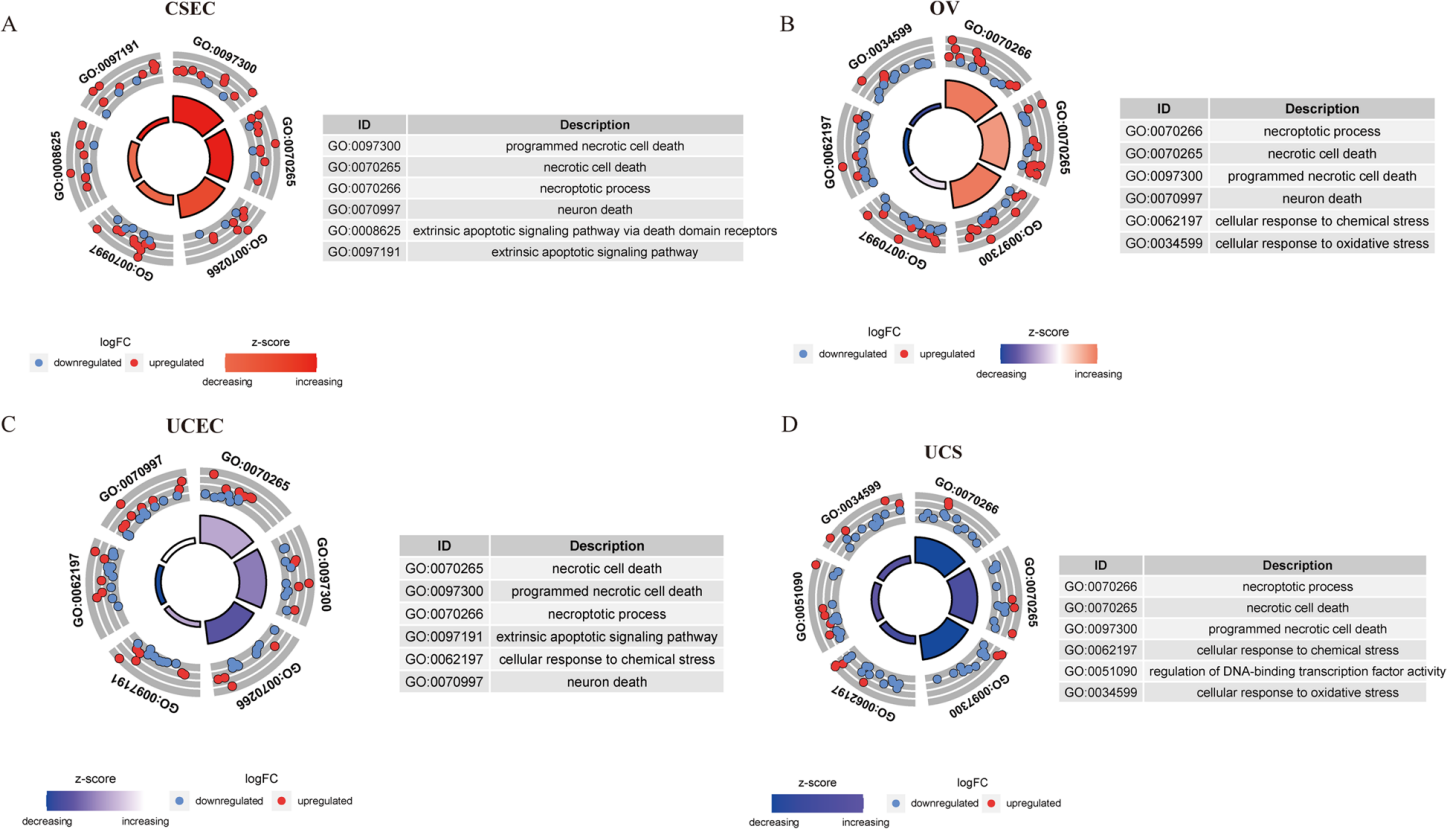
GO enrichment analysis of differentially expressed necroptosis-related genes (NRGs) in the four gynecological cancers (GCs). **A-D** GO enrichment analysis for CESC (**A**), OV (**B**), UCEC (**C**), and UCS (**D**). FC represents fold change. Blue dots indicate genes that were downregulated in the GCs, and red dots indicate genes that were upregulated in the GCs. The size of the z-score is shown by the color

**Fig. 3:**
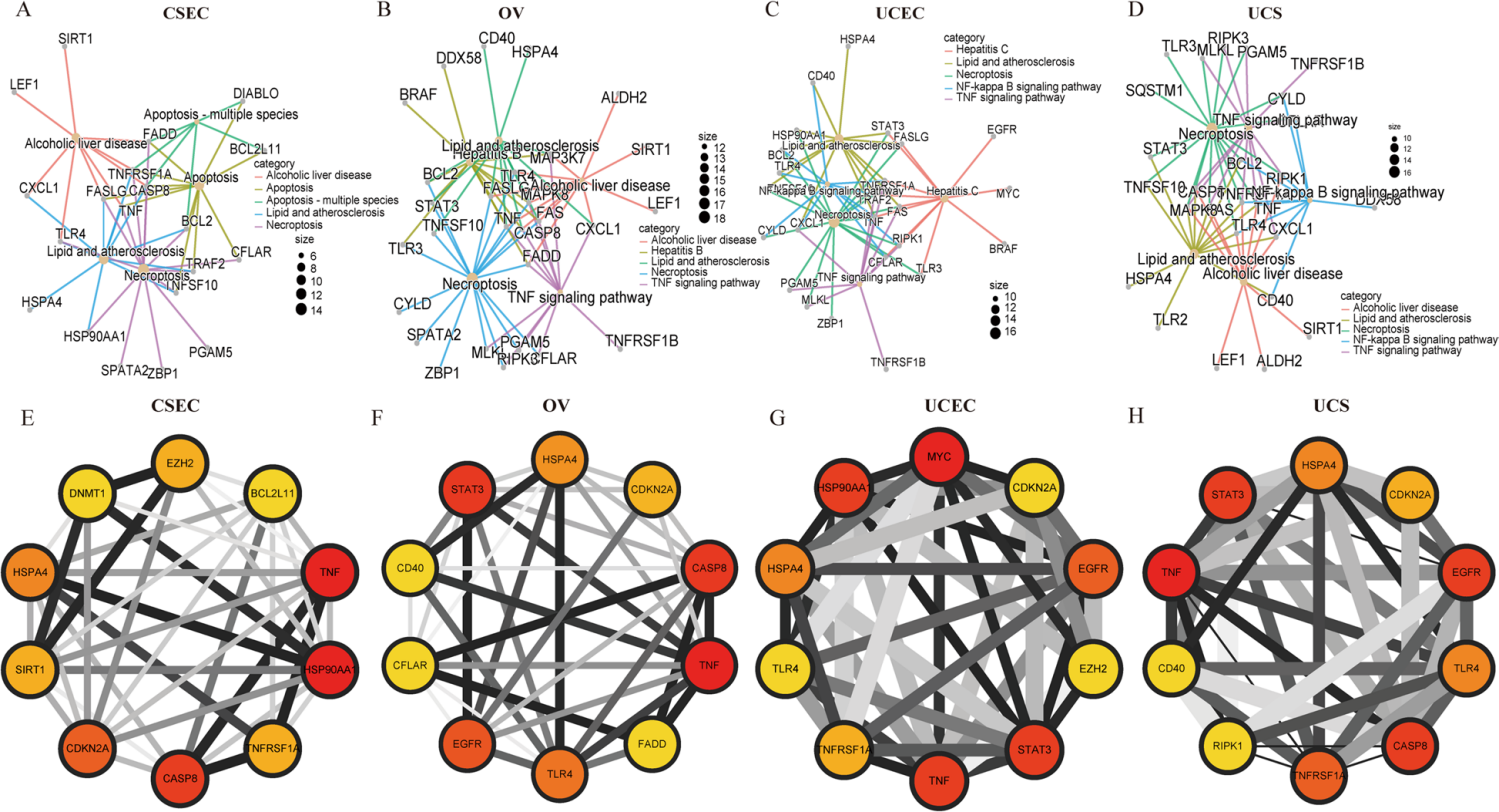
KEGG enrichment analysis and PPI analysis of necroptosis-related genes (NRGs) in the four gynecological cancers (GCs). **A-D** ClueGO results of KEGG analysis of NRGs for CESC (**A**), OV (**B**), UCEC (**C**), and UCS **D**. The size of the dots indicates the number of genes attributed to the category. **E–H** The top 10 hub genes were selected by degree to establish PPI network for CESC (**E**), OV (**F**), UCEC (**G**), and UCS (**H**)

GO enrichment analysis of the four GCs revealed that the differential NRGs were mainly enriched in necroptotic process, necroptotic cell death, and programmed necrotic cell death (Fig. 2A-D). In addition to UCS (Fig. 2D), the differential NRGs of the other three GCs were enriched in neuron dearth. In addition to CESC (Fig. 2C), the differential NRGs of the other three GCs were enriched in cellular response to chemical stress. In addition, the differential NRGs of the GCs were enriched in the same pathway, such as extrinsic apoptotic signaling pathway of CSEC (Fig. 2A) and UCEC (Fig. 2C), and cellular response to oxidative stress of OV (Fig. 2B) and UCS (Fig. 2D). It was interesting to note that extrinsic apoptotic signaling pathway via death domain receptors in OV (Fig. 2A) and regulation of DNA-binding transcription factor activity in UCS (Fig. 2D) were exclusive. The differential NRGs were further selected to construct the PPI network and perform KEGG analysis [65] using Cytoscape software. We demonstrated the top five KEGG pathways in significance, and the results showed that Necroptosis was significantly enriched in all four GCs (Fig. 3A-D). Although not one of top five KEGG pathways in CSEC (Fig. 3A), TNF signaling pathway also showed great value in the other three GCs (Fig. 3B-D). The top ten hub genes were selected by ranking degree. We found that TNF, CDKN2A, and HSPA4 were hub genes in all the four GCs (Fig. 3E-H), indicating their central role in the mechanism study of necroptosis.

**Fig. 4:**
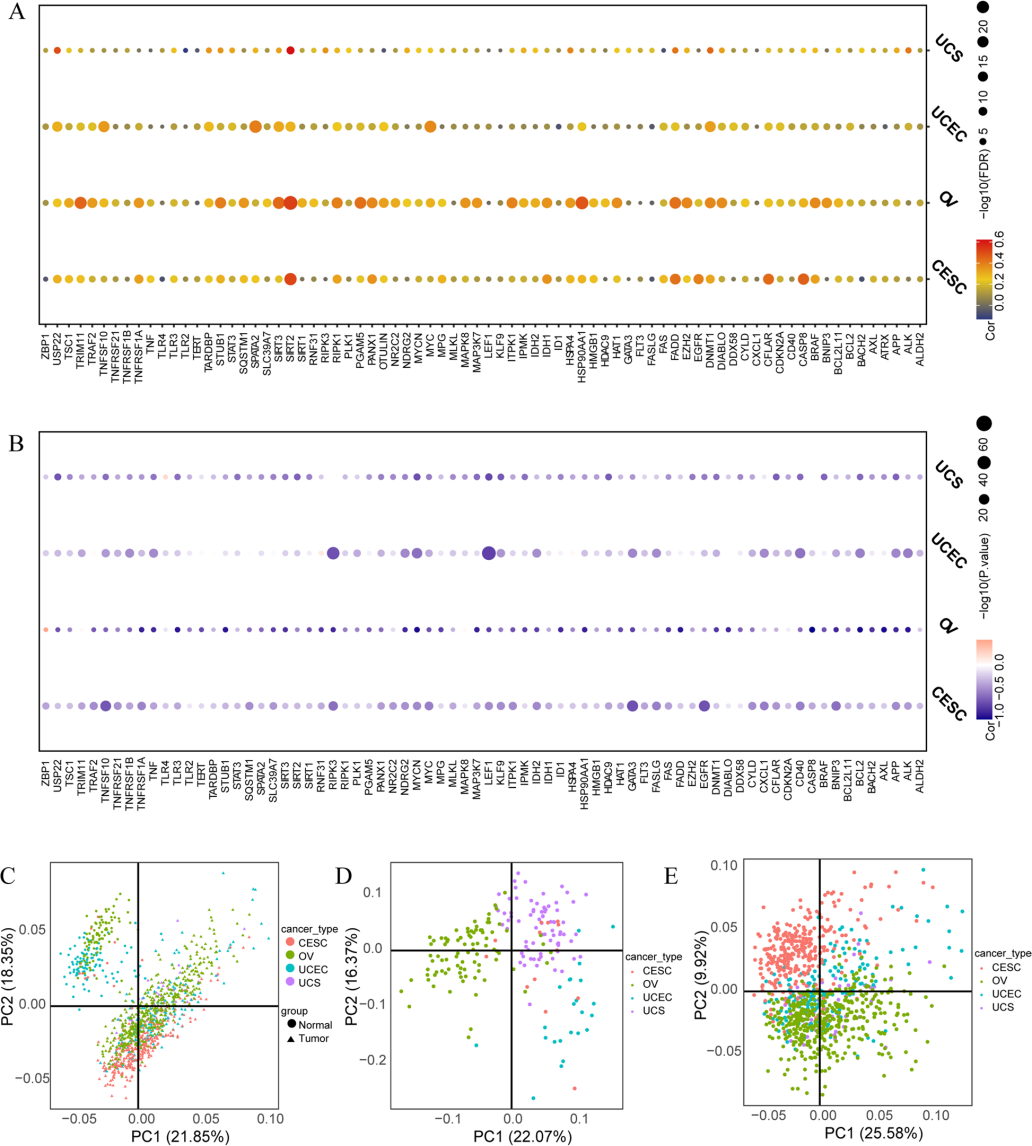
Principal component analysis for necroptosis-related genes (NRGs). **A** Pearson correlation between CNV and NRGs expression level. The bubble color indicates the degree of correlation index. The bubble size indicates the FDR. **B** Spearman correlation between methylation of the NRGs and their expression. The bubble color indicates the degree of correlation index. The bubble size indicates the *P*-value. **C-E** PCA analysis was conducted among all (**C**), normal (**D**), and tumor samples (**E**) respectively. Different gynecological cancers (GCs) are shown in different colors

Among the four GCs, the levels of CNV and NRGs were positively correlated, and the higher association was observed in OV (Fig. 4A). As for the methylation level, most NRGs were negatively correlated (Fig. 4B), which may be a reference for subsequent studies on epigenetic modifications of these NRGs. According to the method, PCA analysis was conducted based on the expression values of the 76 NRGs. Here, PCA analysis was conducted among all (Fig. 4C), normal (Fig. 4D), and tumor samples (Fig. 4E) respectively. It can be seen that no GC showed obvious specific pattern (Fig. 4C-E), while normal and tumor showed obvious separation (Fig. 4C), suggesting the assured distinction between non-cancer and cancer.

The Univariate Cox regression analysis was performed on the NRGs to gain prognostic NRGs. The pan-cancer analysis revealed RNF31 had no prognostic value in any of the 31 tumors (Supplementary Table 3). ALDH2 showed prognostic value in a total of 12 tumors, and was mainly a malignant factor (Supplementary Table 3). The prognostic NRGs were selected for principal component analysis (PCA) for GCs to construct the necroptosis-score. We further carried out Kaplan–Meier analysis to evaluate the survival prognosis of patients with higher necroptosis-score (higher than or equal to the cutoff value) or lower necroptosis-score (lower than the cutoff value). Although it did not reach statistical significance in UCS (Supplementary Figure S3D, *P* = 0.16), our results showed that necroptosis-score showed a tendency to promote GC (Supplementary Figure S3A-D). We also counted the number of differential NRGs and prognostic NRGs in each GC as shown in Supplementary Figure S4.

### Establishment of the prognostic signature based on NRGs

**Fig. 5:**
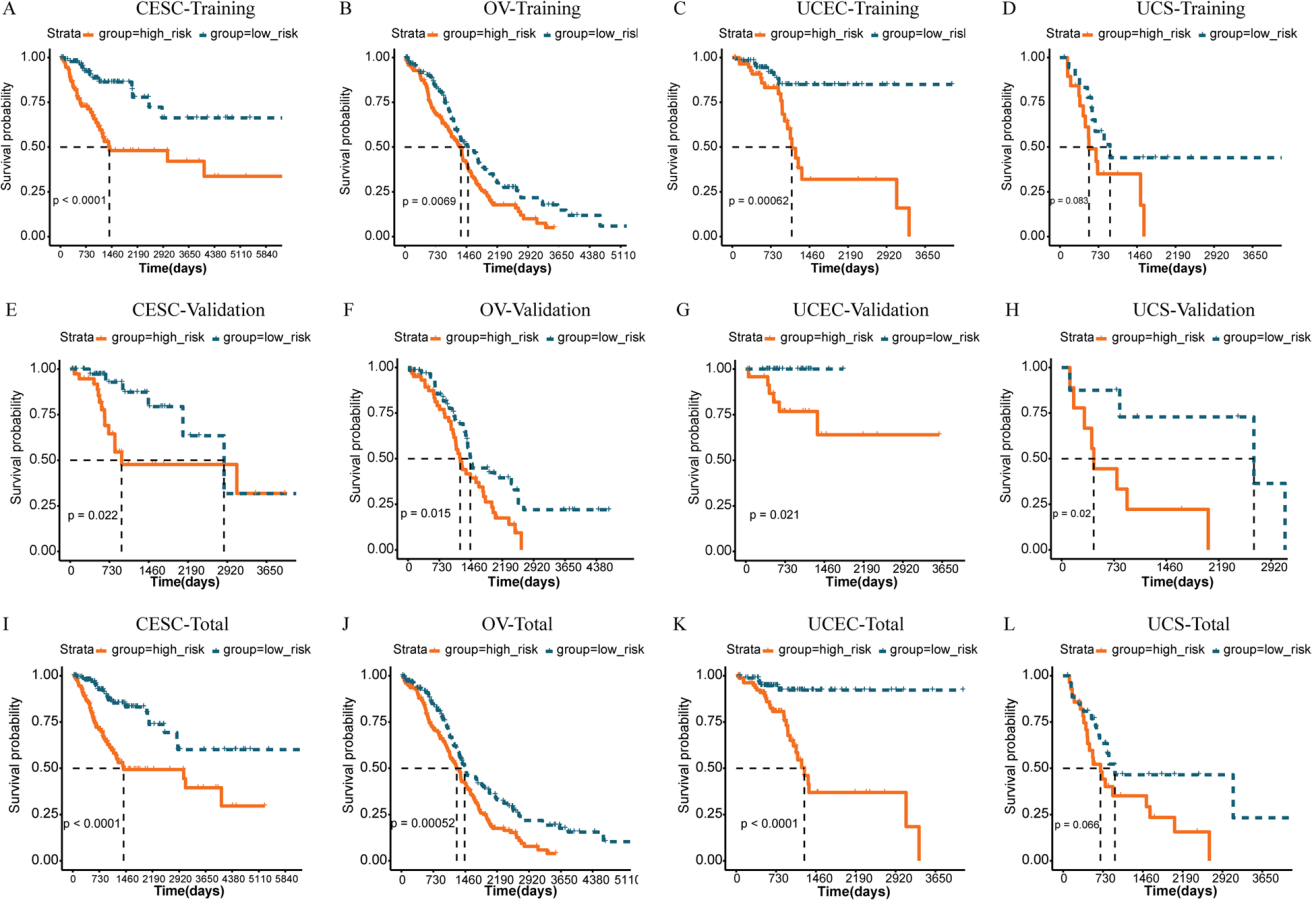
Construction of the prognostic signature based on the optimal NRGs. **A-D** Kaplan–Meier survival curves show survival probability of high-risk or low-risk for CESC (**A**), OV (**B**), UCEC (**C**), and UCS (**D**) in training sets. **E–H** Kaplan–Meier survival curves show survival probability of high-risk or low-risk for CESC (**E**), OV (**F**), UCEC (**G**), and UCS (**H**) in validation sets. **I-L** Kaplan–Meier survival curves show survival probability of high-risk or low-risk for CESC (**I**), OV (**J**), UCEC (**K**), and UCS (**L**) in total sets. The blue curve represents patients in the low-risk group, and the red curve represents patients in the high-risk group

Based on the LASSO regression coefficient of the optimized NRGs screened and the expression level of them in TCGA, we constructed a prognostic model and calculated RSs. The patients from TCGA database were divided into high-risk group (HRG) or low-risk group (LRG) subgroups based on the median value of RSs. Patients with an RS greater than the median RS were placed in HRG, while those with an RS less than the median RS were classified as an LRG. Kaplan–Meier curves revealed that patients in the LRG had good prognosis (Fig. 5A-L). The same results were observed in training, validation, and total sets, which is sufficient to illustrate the accuracy of the prognostic signature we constructed in prognostic prediction. Combined with the survival time and survival status, as well as the RS values of each sample, the ROC curves of 1-year, 3-year and 5-year survival prediction were drawn, showing the promising ability to predict OS (Supplementary Figure S5).

The expression values of five NRGs in cancer tissues and part of normal ovarian tissues in the signature for OV were also determined by qPCR. In TCGA dataset, BACH2, MLKL, MYC and SIRT2 were low expressed in OV tissues, while MYCN was high expressed in OV tissues (Supplementary Figure S6A-E). Similar to the expression differences mentioned above, our data also yielded consistent conclusions. It is worth mentioning that although the difference in the expression value of SIRT2 did not reach statistical significance (*P* = 0.058), the trend was consistent with that in TCGA (Supplementary Figure S6F-J).

**Fig. 6:**
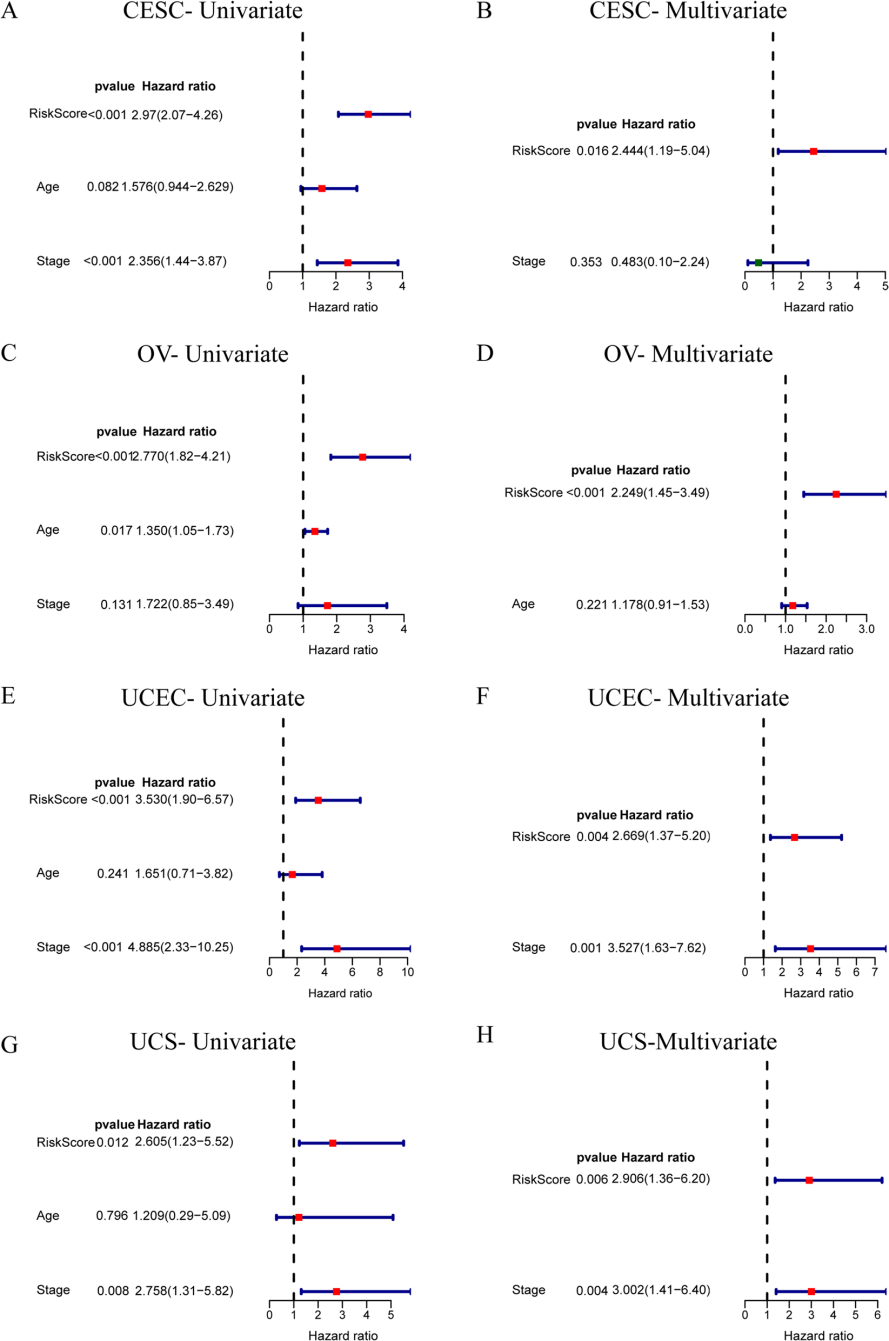
Clinical value of risk score by independent prognostic analysis. **A-H** The Univariate Cox regression analysis and Multivariate Cox regression analysis for CESC (**A-B**), OV (**C-D**), UCEC (**E–F**), and UCS (**G-H**). We reduced the clinicopathological parameters to the ones all GCs shared (Age, Stage)

Considering the inconvenient clinical application of prognostic NRG features in predicting survival in GC patients, a nomogram containing risk groups and clinicopathological parameters was developed. According to the method, we performed Univariate Cox regression analysis for risk group and clinicopathological parameters. The variables with *P* < 0.05 were included in the Multivariate Cox regression analysis, and the variables with *P* < 0.05 were further screened out, as shown in Supplementary Figure S7. The risk model based on NRGs was considered as independent prognostic factors for the four GCs (Fig. 6, Supplementary Figure S7), we reduced the clinicopathological parameters to the ones all GCs shared (Age, Stage, Risk Score) as shown in Fig. 6 for consistency. In addition, it was worth mentioning that Stage was also an independent prognostic factor for UCEC and UCS (Fig. 6F and H). The nomograms were shown in Supplementary Figure S8.

### Evaluation of HALLMARK pathways and mutation between the two risk groups

**Fig. 7:**
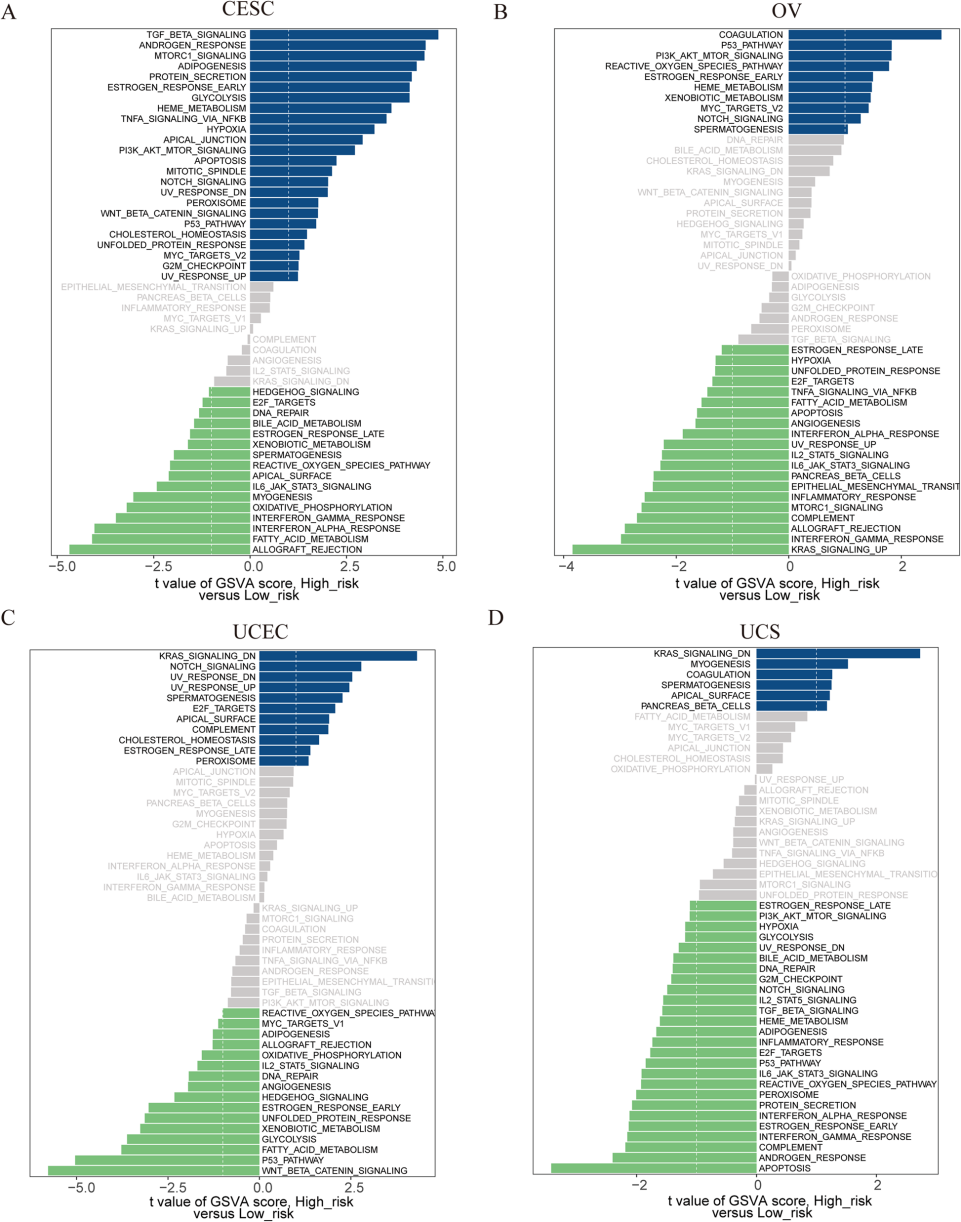
Evaluation of HALLMARK pathways between the two risk groups. **A-D** The bar plots indicate the distribution of the t values of the GSVA scores calculated for several pathways for CESC (**A**), OV (**B**), UCEC (**C**), and UCS (**D**). The blue bars represent HALLMARK pathways that are upregulated in the high-risk group, and the green bars represent HALLMARK pathways that are downregulated in the high-risk group

We first calculated the HALLMARK gene aggregation score of each sample in each GC, and further analyzed the difference between risk groups by using limma package. Unfortunately, we did not find that a single signaling pathways was up- or down-regulated in the HRG of all the four GCs (Fig. 7A-D). Consistent signaling pathways were shown in three of the GCs as follows (Fig. 7A-D): NOTCH SIGNALING (up-regulated, CESC, OV, and UCEC); SPERMATOGENESIS (up- regulated, OV, UCEC, and UCS); ALLOGRAFT REJECTION (down-regulated, CESC, OV, and UCEC); DNA REPAIR (down-regulated, CESC, UCEC, and UCS); E2F TARGETS (down-regulated, CESC, OV, and UCS); ESTROGEN RESPONSE LATE (down-regulated, CESC, OV, and UCS); FATTY ACID METABOLISM (down-regulated, CESC, OV, and UCEC); IL2 STAT5 SIGNALING (down-regulated, OV, UCEC, and UCS); IL6 JAK STAT3 SIGNALING (down-regulated, CESC, OV, and UCS); INTERFERON ALPHA RESPONSE (down-regulated, CESC, OV, and UCS); INTERFERON GAMMA RESPONSE (down-regulated, CESC, OV, and UCS); REACTIVE OXYGEN SPECIES PATHWAY (down-regulated, CESC, UCEC, and UCS). There were many more pathways that showed consistency between the two GCs. The distribution variations of the somatic mutations between the two risk groups were also analyzed. The top 20 mutated genes in the HRG and LRG were consistent in the four GCs (Supplementary Figure S9). We found that TTN had the highest mutation frequency in CESC (Supplementary Figure S9A-B), TP53 in OV (Supplementary Figure S9C-D) and UCS (Supplementary Figure S9G-H), and PTEN in UCEC (Supplementary Figure S9E-H).

### Evaluation of immune activity and immunotherapy between the two risk groupsactivated/Mast cells resting

**Fig. 8:**
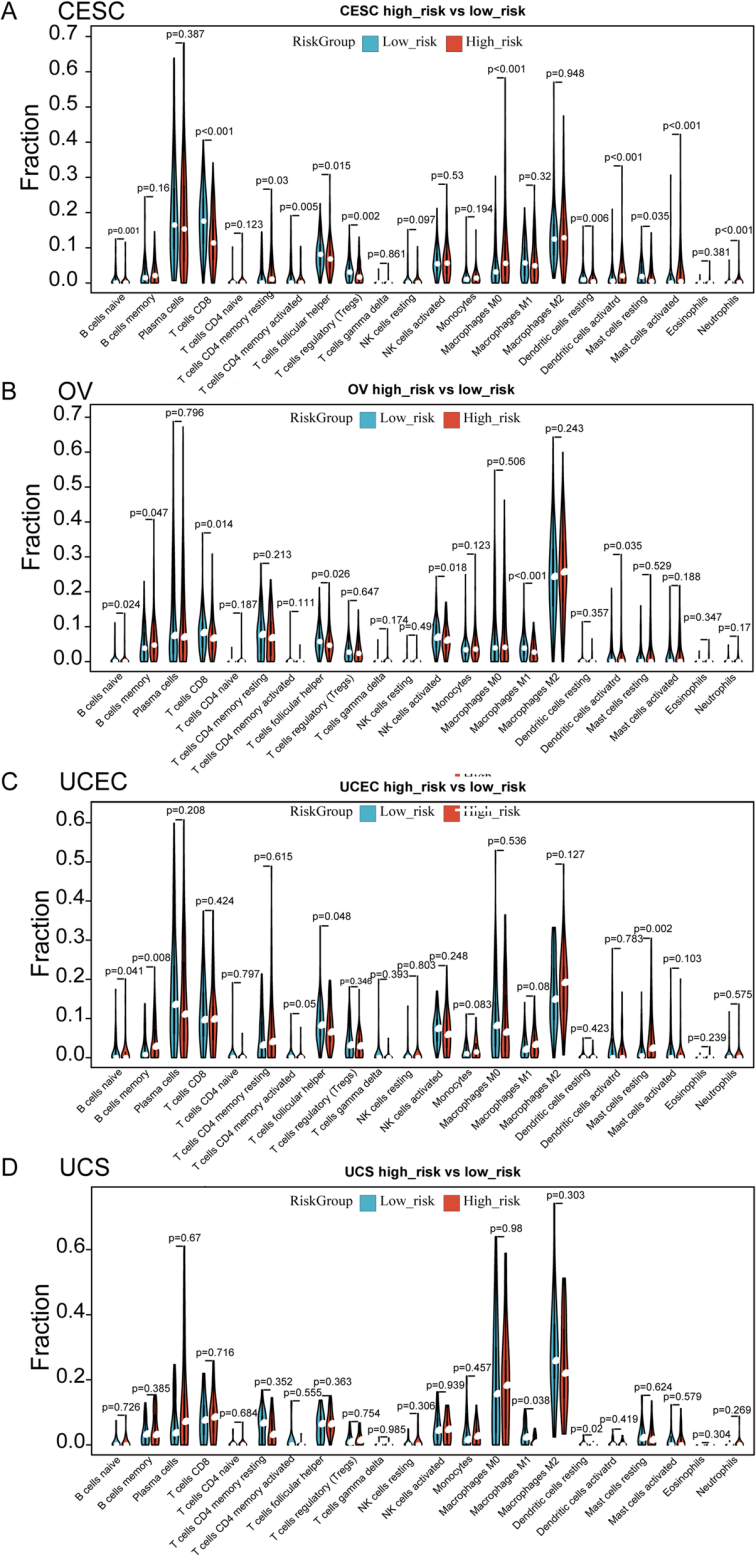
Evaluation of immune activity between the two risk groups. **A-D** The distribution of 22 different immune cells between high and low risk groups for CESC (**A**), OV (**B**), UCEC (**C**), and UCS (**D**). The blue violins represent the low-risk group, and the red violins represent the high-risk group

To further explore the relationship between immune activity and NRG-signature, CIBERSORT was used to analyze immune activity between the two risk groups. We found that except for UCS, B cells naive and T cells follicular helper were different between the high- and low-risk groups of CESC, OV and UCEC **(**Fig. 8A-D). There was clearly heterogeneity in the immune microenvironment of individual GCs, as more immune cells showed differences in only one GC, such as T cells CD8/T cells CD4 memory resting/T cells CD4 memory activated/T cells regulatory (Tregs)/ Macrophages M0/Dendritic cells resting/Dendritic cells activated/Mast cells resting/Mast cells activated/Neutrophils in CESC (Fig. 8A), B cells memory/T cells CD8/NK cells activated/Macrophages M1/Dendritic cells activated in OV (Fig. 8B), B cells memory/Mast cells resting in UCEC (Fig. 8C), Macrophages M1/Dendritic cells resting in UCS (Fig. 8D). Therefore, the prognostic signature of CESC could better distinguish the immune microenvironment, while UCS was poor in this respect. Further, for each GC, spearman correlation coefficient between 22 immune cells was calculated, and the relationship of *P* < 0.05 was displayed by bubble heat map to observe the differences among the four GCs (Supplementary Figure S10), and it was observed that cervical and ovarian cancer showed more correlation.

**Fig. 9:**
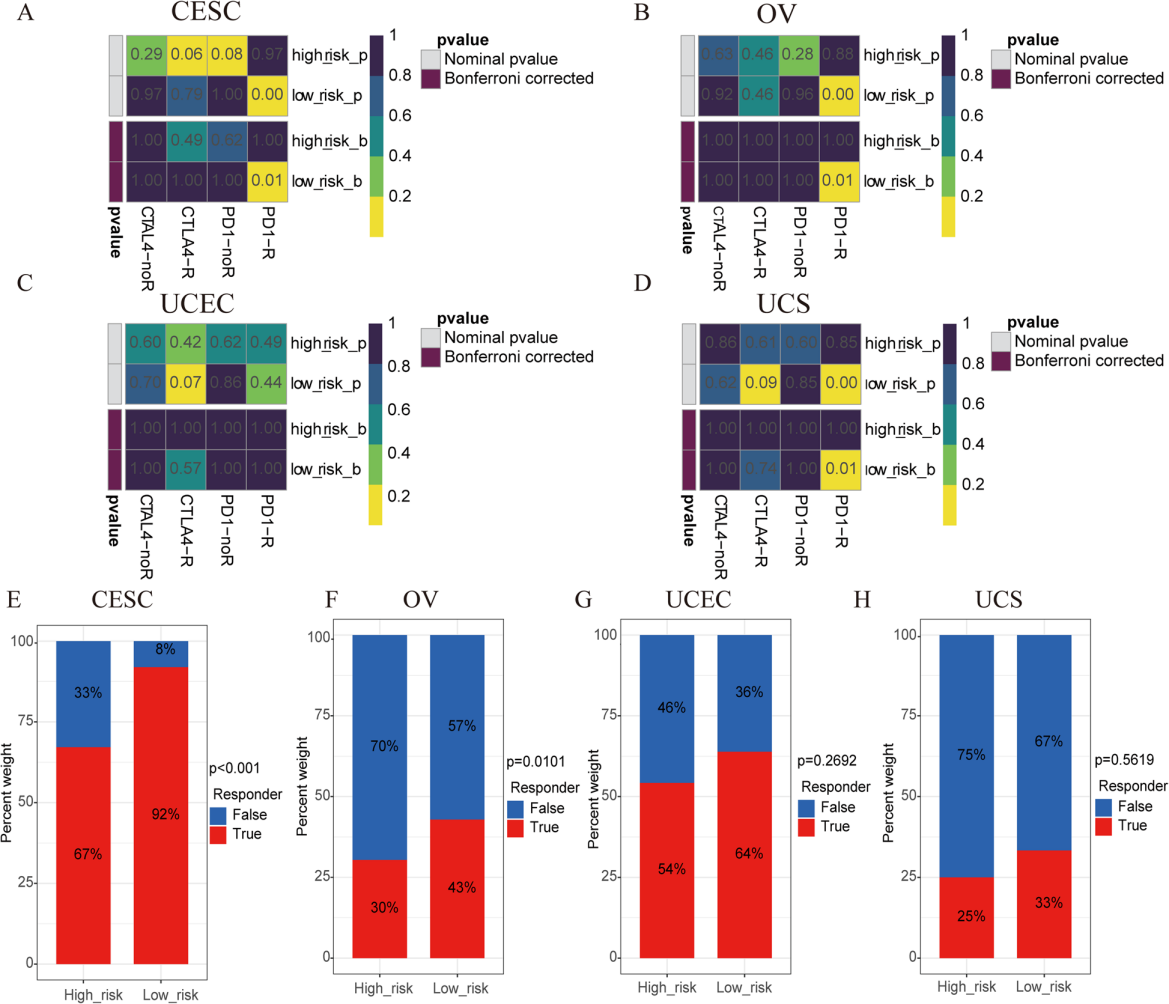
Evaluation of immunotherapy between the two risk groups. **A-D** The subclass mapping for CESC (**A**), OV (**B**), UCEC (**C**), and UCS **D**. **E–H** The ICB response rates for CESC (**E**), OV (**F**), UCEC (**G**), and UCS **H**

Furthermore, we investigated the association between our risk model and immune checkpoints (Supplementary Figure S11). Among them, CD200R1 and CD44 were statistically different in CESC, UCEC, and UCS, while CD244 and CTLA4 were statistically different in CESC, OV, and UCEC (Supplementary Figure S11). Through the results of subclass mapping, we found that GC patients in the LRG may be more sensitive to PDL1 response, except for UCEC (Fig. 9A-D). In line with this, the ICB response rates were higher in the LRG, suggesting that GC patients with low RSs are more sensitive to immune checkpoint blockade therapy (Fig. 9E-H).

**Fig. 10:**
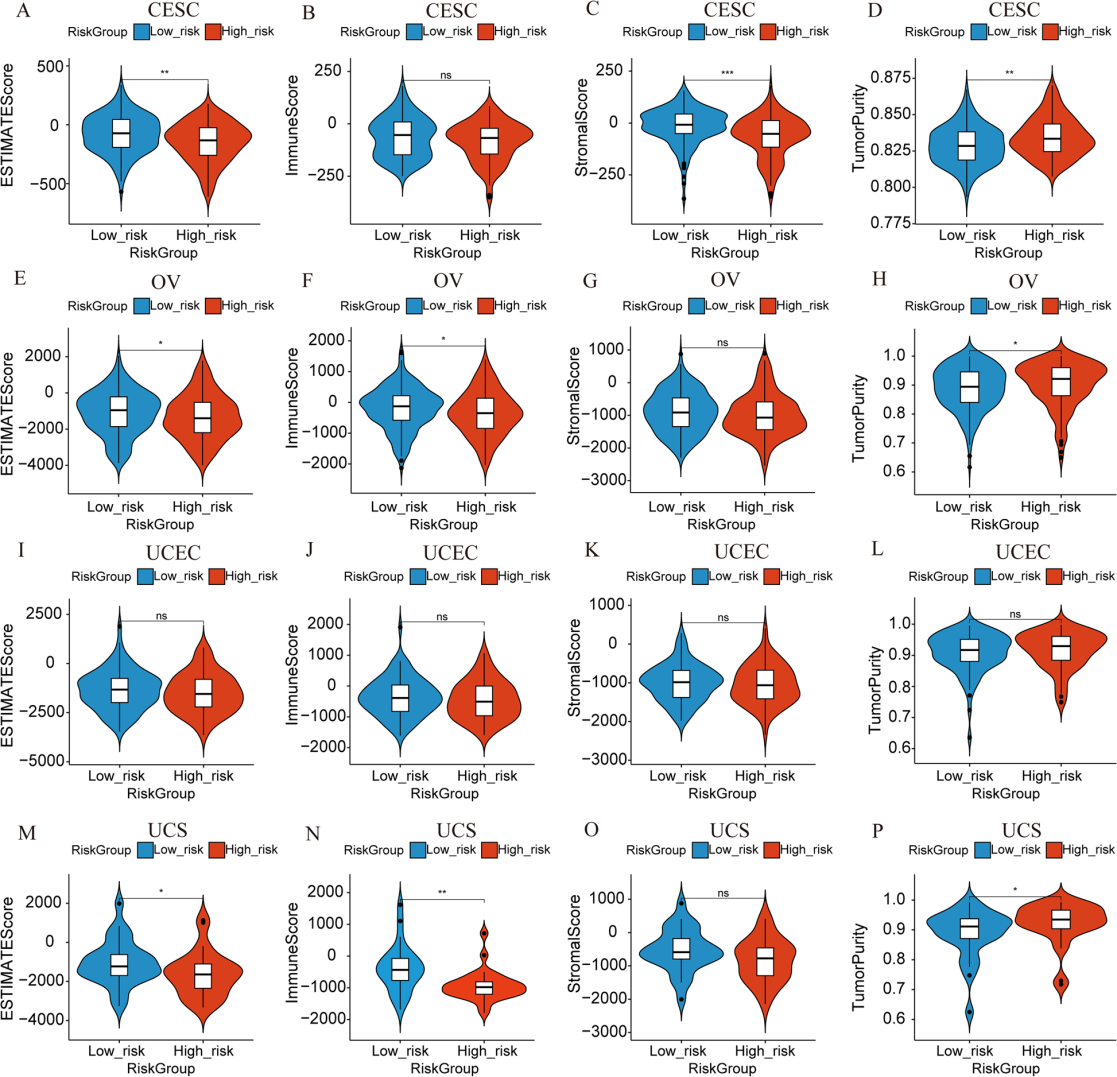
Evaluation of estimate-related scores between the low-risk and high-risk groups. **A-D** Comparison of Estimate Score (**A**), Immune Score (**B**), Stromal Score (**C**) and Tumor Purity (**D**) for CESC. **E–H** Comparison of Estimate Score (**E**), Immune Score (**F**), Stromal Score (**G**) and Tumor Purity (**H**) for OV. **I-L** Comparison of Estimate Score (**I**), Immune Score (**J**), Stromal Score (**K**) and Tumor Purity (**L**) for UCEC. **M-P** Comparison of Estimate Score (**M**), Immune Score (**N**), Stromal Score (**O**) and Tumor Purity (**P**) for UCEC. ∗ *P* < 0.05; ∗  ∗ *P *< 0.01; ∗  ∗  ∗ *P* < 0.001; ns: not significant. The blue violins represent the low-risk group, and the red violins represent the high-risk group

Subsequently, we estimated immune score, stromal score, ESTIMATE score, and tumor purity using ESTIMATE algorithm, thus comparing their distinction between the risk groups (Fig. 10). On average, the GC patients in the LRG had higher ESTIMATE score (Figure A, E, I, and M), immune score (Fig. 10 B, F, J, and N) and stromal score (Fig. 10 C, G, K, and O), while GC patients with higher RSs had higher tumor purity (Fig. 10 D, H, L, P). From the overall trend, the higher the ESTIMATE score /immune score /stromal score, the lower the tumor purity, which was consistent with earlier study [60]. These score differences may also partly explain why low-risk patients were more responsive to immunotherapy and had a better prognosis.

### Drug susceptibility analysis

**Fig. 11:**
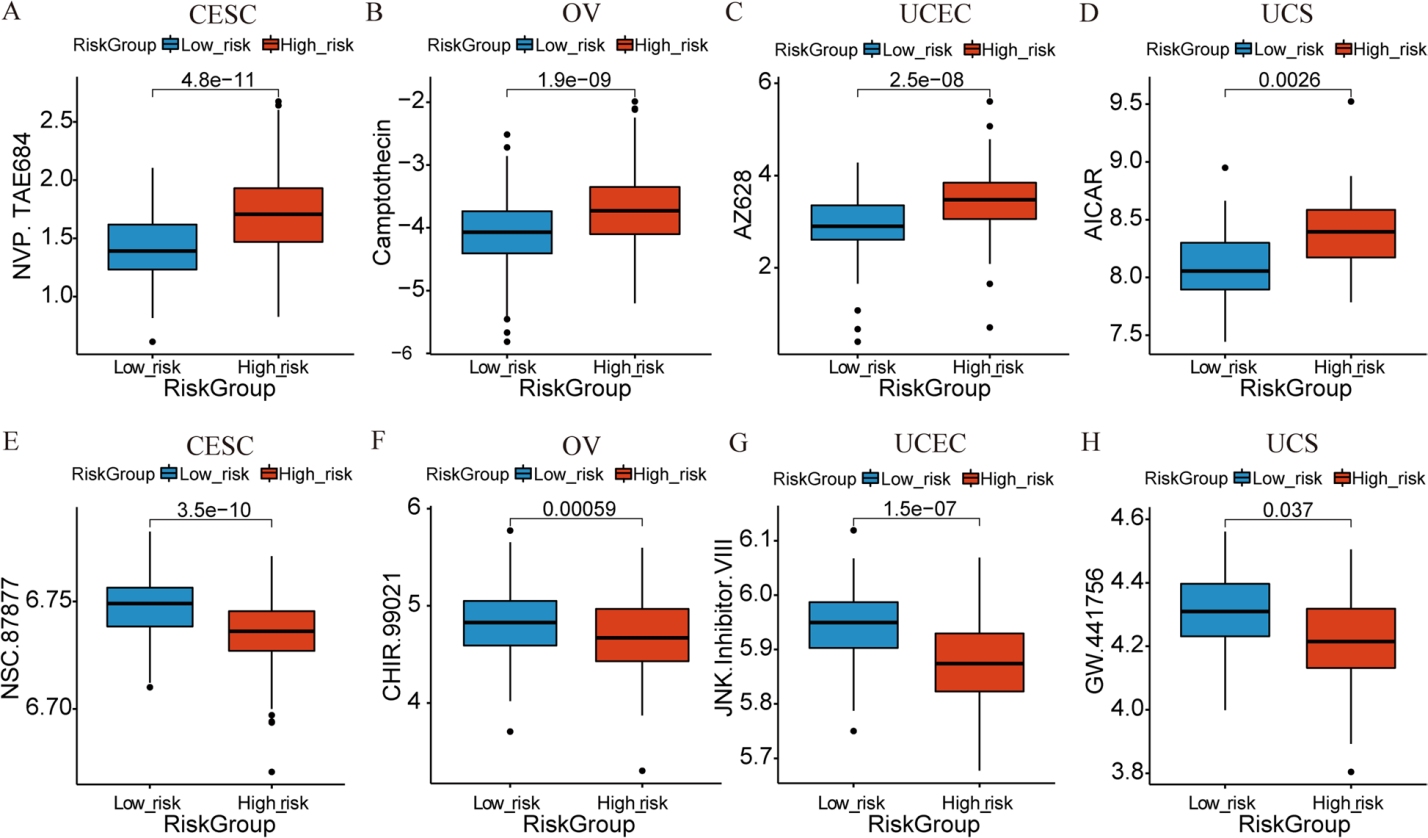
Analysis of chemotherapeutic sensitivity based on the NRG-signature. **A-H** Relationships between risk scores and IC50 level of drugs. Only the drug with the most significant IC50 difference in each tumor was shown. **A-D** Drugs with IC50 values most significantly higher in the high-risk group for CESC (**A**), OV (**B**), UCEC (**C**), and UCS (**D**). **E–H** Drugs with IC50 values most significantly higher in the low-risk group for CESC (**E**), OV (**F**), UCEC (**G**), and UCS (**H**). The blue bars represent the low-risk group, and the red bars represent the high-risk group

According to the method, based on the drugs provided by pRRophetic package and combined with the gene expression value, we estimated the IC50 value of each drug, and further compared the difference between the two risk groups. The results showed that, 51, 45, 64, and 29 drugs with differences between risk groups were yielded in CESC, OV, UCEC, and UCS respectively. Only the drug with the most significant IC50 difference in each tumor (higher RS or lower RS) was shown in Fig. 11A-H. From these drugs with IC50 values that differ between risk groups, we may be able to target different treatment regimens to different patients with GC. According to the method, spearman correlation coefficients between NRGs in NRG-signature of each GC and drugs with significant differences in IC50 obtained above were calculated respectively. According to the threshold setting, only significant relationship pairs with absolute correlation coefficients greater than 0.3 were shown in Supplementary Figure S12. We can see that most of them are negatively correlated.

## Discussion

The main ways of cell death include autophagy, apoptosis, necrosis, ferroptosis, oncosis, paraptosis, necroptosis and so on [66]. In the past, apoptosis was considered to be the only programmed process of cell death, while cell necrosis was considered to be an uncontrollable passive biological behavior. However, recent studies have shown that necroptosis is also regulated by intracellular signaling pathway networks [8, 67]. Necroptosis is a different way of cell death from apoptosis. It often does not depend on Caspase, the key regulator of apoptosis, and can mediate cell death when apoptosis is inhibited. It has necrotic cell morphology and loss of cell membrane integrity is often present, which can be inhibited by the specific small molecule Nec-1 [67]. Many studies have shown that necroptosis is often inhibited during the genesis and development of tumor cells. For example, in chronic lymphocytic leukemia cells, RIP3 and CLYD, which are important regulators of necroptosis, are significantly down-regulated [68]. However, it has also been proved that necroptosis plays a dual role in cancer progression and development [12]. Among them, targeted necrosis proteins have dual effects on the occurrence and development of tumors [14]. Due to the dual role of necroptosis in tumor development and antitumor therapy, it is necessary to further explore the exact molecular mechanisms of the key molecules of necroptosis and their interactions with other proteins, especially MLKL, the executor of necroptosis and its downstream unknown structures. MLKL protein is widely found in many human organs and low expression in many tumor tissues, such as brain tumors [69]. Through differential analysis of NRGs in pan-GCs, we found that MLKL was low expressed in OV, UCEC, and UCS, and the low expression of MLKL in OV was also confirmed in the samples we collected. Ulteriorly, through Univariate Cox and LASSO Cox regression analyses, we found that MLKL may be more important for the occurrence and development of OV, since it is involved in the construction of the prognostic NRG-signature of OV. Compared with normal control tissue, other common differential NRGs in pan-GCs were also found in our study, including up-regulated NRGs (CDKN2A, CXCL1, DIABLO, EZH2, GATA3, HSPA4, IDH2, PGAM5, PLK1, TERT, TNF, and TNFRSF21) and low-expressed NRGs (ATRX, AXL, BACH2, BCL2, BRAF, CFLAR, KLF9, NDRG2, NR2C2, SIRT1, SIRT3, TLR4, TSC1, USP22). In the subsequent PPI network, we were surprised to observe that TNF, CDKN2A, and HSPA4 were the hub genes of the four GCs. Most of the current understanding of the molecular mechanism of necroptosis comes from the study of TNF-induced necroptosis signaling pathway [70]. Although CDKN2A and HSPA4 are also the necroptosis-related proteins, there are still few studies on it [33, 71]. Hence, these common differential NRGs play an important role in female reproductive system tumors, but the specific mechanism still needs to be further studied. Some of the differential NRGs are unique to a particular gynecologic tumor, such as BCL2L11 and OTULIN in CESC, MAP3K7 in OV, TLR2 in UCS. Interestingly, the expression status of RNIP3, CASP8, DDX58, FLT3, HDAC9, ID1, LEF1, RIPK3, TLR3, and TNFSF10 (high or low expression) was heterogeneous among the four tumors, indicating the dual role of NRGs in gynecological tumors to a certain extent. Ulteriorly, we performed enrichment analysis on the differential NRGs of various GCs, and the results showed that these NRGs were indeed significantly enriched in necroptotic process, necroptotic cell death, programmed necrotic cell death and necroptosis which further confirmed the important role of these genes in necroptosis. However, the enrichment pathways of the four GCs were also different to some extent, as explained in the RESULTS section. The common characteristics or differences in enrichment pathways can provide references for subsequent mechanism verification.

Current studies believe that CNV is not only the basis of individual genetic differences, but also plays an important role in somatic malignant transformation, tumorigenesis, progression and metastatic colonization [72]. CNV related indicators may become ideal tumor diagnostic markers. Our data indicated that the levels of CNV and NRGs were positively correlated among the four GCs in the mass. Methylation is an important modification of proteins and nucleic acids that regulates the expression and shutdown of genes and is closely related to cancer [73]. In our study, most NRGs were negatively correlated with methylation level, which may be a reference for subsequent studies on epigenetic modifications of these NRGs.

Necroptosis, as a newly discovered mode of death, has attracted more and more attention in the field of gynecologic tumor. The induction of necroptosis was observed in ovarian cancer and the expression of catalytically active RIPK3 (receptor-interacting protein kinase-3) was necessary for death [23]. RIPK3 expression status could critically influence immunotherapy of cervical cancer [24, 25]. Li Liu et al. found that necroptosis induced by the combination therapy of Berberine and Cisplatin can kill ovarian cancer cells and improve treatment [26]. Two ALDH1A family selective inhibitors (ALDH1Ai) were identified to overcome chemotherapy resistance and outcomes of ovarian cancer through the necroptotic death of CSCs (cancer stem-like cells) [27]. It was reported that RIP1 could mediate cisplatin-induced necroptosis [28]. Xuewei Zhang et al. found that ceramide nanoliposomes could serve as necroptosis-inducing chemotherapeutic reagent [29]. Necroptosis was identified to be the main anticancer mechanism of CuS-MnS2 nano-flowers + NIR [74]. A novel process-enzyme-instructed self-assembly (EISA) could also cause necroptosis to kill ovarian cancer cell [75]. DEBIO 1143, a SMAC (second mitochondria-derived activator of caspase) mimetic was able to reverse carboplatin resistance by necroptosis and potentiate carboplatin treatment [30]. Caspase8 could promote necroptosis by stabilizing RIPK1 [31] and necroptosis was reported to be induced by RETrA (REactivation of Transcriptional Reporter Activity) through the phosphorylation of RIPK1 and RIPK3 thus playing a therapeutic role in cervical cancer [76]. It was reported that BMI1 [33] or ARHI (DIRAS3) [34] could induce autophagy-mediated necroptosis and necroptosis could augment tumor-associated macrophages M1 polarization [35] and autophagy through other pathways [36, 37]. Xiaofeng Chen et al. found that Youdujing extract could promote the combination of RIP1 with RIP3 and MLKL to facilitate necroptosis [38]. Further study of the regulation mechanism of necroptosis and blocking revulsant, is not only beneficial to deepen the understanding and awareness of cell death, and is helpful to the research of different diseases. The relationship between necroptosis and cancer will continue to become a research hotspot a long time in the future. In-depth study on the role of necroptosis in the pathogenesis of GCs will promote the development of new therapeutic targets and provide valuable clues and means for the research and development of related molecular target drugs. Obviously, necroptosis has great potential in tumor research, which is worthy of continuous exploration and in-depth study. At present, the preliminary studies mainly focus on ovarian cancer, while the research on UCEC and UCS is still blank. Based on PCA analysis, we found that necroptosis-score showed a tendency to make the prognosis of GCs worse. It is worth noting that although this tendency was also shown in UCS, it did not reach statistical significance, which may be due to the scarcity of samples in UCS. According to the existing studies, the induction of necroptosis can promote the development of breast cancer [77] and pancreatic ductal adenocarcinoma [13]. However, evidence for the role of necroptosis in GCs is still lacking, therefore, the net effect of necroptosis in GCs needs to be conclusively determined.

Screening and risk assessment of GCs are very important for the diagnosis and treatment of diseases. With the development of molecular biology, more studies are devoted to screening polygenes as prognostic biomarkers. In this study, we carried out LASSO Cox regression analysis and then we established the prognostic NRG-signature based on corresponding poly-NRGs. The survival curves showed the significantly worse clinical outcomes of patients with higher risk-scores (RSs) while the prognosis of patients with lower RSs was better. The same results were observed in training, validation, and total sets, which is sufficient to illustrate the accuracy of the prognostic signature we constructed in prognostic prediction. The AUCs of 1-year, 3-year, and 5-year survival ROC curves predicted by the NRG-signature were large, suggesting the efficiency of NRG-signature in predicting prognosis for GCs. Furthermore, NRG-signature was an independent prognostic factor for GCs demonstrated by Univariate and Multivariate Cox regression analyses. In a word, the prognosis and independent prognostic value of the NRG-signature was determined for the four GCs sufficiently. Our study was the first to report NRG-signature in pan-GC and we performed extensive analyses to identify signature and its biological implications. Although there have been articles published on prognostic signature of one type of GC, all prognostic features were different and there is no uniform prognostic feature in clinical practice. Other studies were mainly based on one GC, and the pan-GC analysis based on necroptosis we conducted can more intuitively reflect the commonality and heterogeneity among gynecologic tumors. As for genes in the prognostic NRG-signature, three genes (DNMT1, MYC, and SIRT2) could be used to construct prognostic signatures for CESC and OV, revealing the great value of these three genes in predicting the prognosis of CESC and OV. The remaining NRG-signature genes showed outstanding prognostic value in their respective tumors. Therefore, the prognostic characteristics of the four GCs were both common and heterogeneous.

To further clarify when necroptosis exerts its antitumor effect, how to regulate necrotic anti-tumor therapy, and maximize the antitumor effect of necroptosis, tumor cell types and tumor microenvironment (TME) and other factors should be considered, so as to provide new targets for tumor targeted therapy. More importantly, it is necessary for us to select appropriate schemes to induce different forms of cell death of tumor cells according to tumor cell types, combined with chemotherapy, radiotherapy and immunotherapy, so as to jointly exert anti-tumor effects, and explore the internal relationship and coordination mechanism between different types of cell death. In recent years, it has been found that many compounds and anticancer drugs can induce tumor cells to produce necroptosis in various ways, thus killing tumor cells [78–80]. For example, a sphinolipid ceramide analogue, FTY720, has been found to induce necroptosis in tumor cells. This effect depends on the intraconuclear binding of FTY720 with I2PP2A/SET tumor protein to release inhibition of tumor suppressor protein PP2A protein, thereby activating its expression [81]. Tumor therapy based on necroptosis is a new strategy for anti-tumor therapy, but its feasibility is still controversial. Proponents believe that because necroptosis and apoptosis function through different signaling pathways, inducing necroptosis of tumor cells has potential as an alternative therapy for anti-apoptotic malignancies. According to the current research, this hypothesis has been preliminarily verified. However, skeptics argue that congenital or acquired defects in the mechanism of necrosis have been observed in many cancer cells, and further research remains to be done on whether the use of necrosis inducers can selectively kill cancer cells without interfering with normal cell activity and whether they lead to deinflammatory effects in vivo. Herein, we explore the relationship between TME (mainly immune activity) and NRG-signature, our results showed that except for UCS, B c©naive and T cells follicular helper were different between the high- and low-risk groups of CESC, OV and UCEC. As for immunotherapy, we observed that GC patients with lower RSs may be more sensitive to PDL1 response and immune checkpoint blockade therapy. In addition, the sensitivity of chemotherapeutic agents to prognostic signatures and NRGs of four gynecologic tumors was also investigated, which may provide preliminary evidence for future therapies targeting necroptosis.

We carried out comprehensive pan-cancer analysis of necroptosis molecules in four gynecologic cancers, thus evaluating the similarities and differences in necroptosis. Furthermore, our study proposed new prognostic signature based on necroptosis for GCs, whose clinical applicability deserves further exploration. Our study still has some limitations. The analytical data were from TCGA, hence, we need use the tissues we collected to verify this model in subsequent studies and closely follow GC patients. Due to the lack of samples, we only detected the expression values of several NRGs in the prognostic signature of OV. Since the samples were recently collected, further follow-up is needed to determine the outcome of the OV patients, thus further verifying the accuracy of this signature in predicting prognosis. Due to the lack of time and money for follow-up, the specific mechanism of NRGs identified by us has not been developed yet. Currently, we are committed to the study of mRNAs based on the regulation of necroptosis in GCs, so as to overcome the clinical thorny problem of difficult diagnosis and poor prognosis of GCs.

## Conclusions

Our integrative analysis of necroptosis molecules revealed a broad regulatory mechanism affecting clinicopathological features, immune activity and prognosis. Furthermore, we identified the therapeutic responsibility of necroptosis-related genes in immunotherapy and targeted therapy. These features were well compared in four gynecologic cancers. These findings demonstrate the important clinical significance of necroptosis-related genes, and will afford new thoughts to direct the personalized immunotherapy strategy for patients with gynecologic cancers.

## Supplementary Information

**Additional file 1: Supplementary Figure S1.** Mutation frequency and expression variation of the 76 necroptosis-related genes (NRGs). **A-D** Mutation frequency of NRGs in patients with CESC (**A**), OV (**B**), UCEC (**C**), and UCS **D**. The small figure above shows the TMB, the number on the right shows the mutation frequency of each NRG, and the figure on the right shows the proportion of each vari©. **E** Expression levels of NRGs in four gynecological tumors. The color of the dots represents the degree of variance. Redder dots represent higher expression in cancer tissue. Bluer dots represent higher expression in normal tissue. The size of the bubbles indicates the adjusted *P*-value. Larger bubbles represent a lower adjusted *P*-value. The genes with adjusted *P*-value<0.05 & |logFC|>0.5 were retained to produce the figure.

**Additional file 2: Supplementary Figure S2.** Correlation analysis of the 76 NRGs for CESC (**A**), OV (**B**), UCEC (**C**), and UCS **D**. Red indicates a positive correlation; blue indicates a negative correlation.

**Additional file 3: Supplementary Figure S3.** The survival curves of necroptosis-score. **A-D** Kaplan–Meier curve was used to analyze the survival rate of CESC (**A**), OV (**B**), UCEC (**C**), and UCS (**D**) patients with high or low necroptosis-score. Black curves indicate low necroptosis-score and red curves indicate high necroptosis-score.

**Additional file 4: Supplementary Figure S4.** Statistical analysis of the number of differential NRGs and prognostic NRGs of the four GCs. Red bars show up-regulated NRGs in cancer tissue. The green bars show NRGs that are downregulated in cancer tissue. The blue bars show the sum of the differential NRGs. Yellow bars indicate prognostic NRGs.

**Additional file 5: Supplementary Figure S5.** The ROC curves for the risk model in the four GCs. **A-C** The ROC curves of CESC for training (**A**), validation (**B**), and total (**C**) sets. **D-F** The ROC curves of OV for training (**D**), valida©n (**E**), and total (**F**) sets. **G-I** The ROC curves of UCEC for training (**G**), validation (**H**), and total (**I**) sets. **J-L** The ROC curves of UCS for training (**J**), validation (**K**), and total (**L**) sets.

**Additional file 6: Supplementary Figure S6.** Expression values of NRGs in prognostic signature of OV. **A-D** Expression values of NRGs in TCGA for BACH2 (**A**), MLKL (**B**), MYC (**C**), MYCN (**D**), and SIRT2 (**E**). **F-J** Expression values of NRGs in our cohort for BACH2 (**F**), MLKL (**I**), MYC (**G**), MYCN (**H**), and SIRT2 (**J**).

**Additional file 7: Supplementary Figure S7.** Clinical value of risk score by independent prognostic analysis. **A-H** The Univariate Cox regression analysis and Multivariate Cox regression analysis for CESC (**A-B**), OV (**C-D**), UCEC (**E-F**), and UCS **G-H**.

**Additional file 8: Supplementary Figure S8.** The Nomogram model based on risk model and clinical features for GCs. **A-B** The Nomogram (**A**) and calibration curve (**B**) for CESC. **C-D** The Nomogram (**C**) and calibration curve (**D**) for OV. (**E-F**) The Nomo©m (**E**) and calibration curve (**F**) for UCEC. **G-H** The Nomogram (**G**) and calibration curve (**H**) for UCS.

**Additional file 9: Supplementary Figure S9.** The waterfall plot of somatic mutation features established with risk scores. **A-B** The waterfall plot of somatic mutation in CESC for high-risk group (**A**) and low-risk group **B**. **C-D** The waterfall plot of somatic mutation in OV for high-risk group (**C**) and low-risk group **D**. **E-F** The waterfall plot of somatic mutation in UCEC for high-risk g©p (**E**) and low-risk group **F**. **G-H** The waterfall plot of somatic mutation in UCS for high-risk group (**G**) and low-risk group **H**.

**Additional file 10: Supplementary Figure S10.** Spearman correlation coefficient between 22 immune cells was calculated for CESC (**A**), OV (**B**), UCEC (**C**), and UCS **D**. Red bubbles indicate positive correlations and blue bubbles indicate negative correlations. The numbers in the bubbles represent the correlation coefficients.

**Additional file 11: Supplementary Figure S11.** Expression of immune checkpoints in the low and high-risk groups for CESC (**A**), OV (**B**), UCEC (**C**), and UCS **D**. ∗*P*<0.05; ∗∗*P*<0.01; ∗∗∗*P*<0.001; ns: not significant. The blue bars represent the low-risk group and the red bars represent the high-risk group.

**Additional file12: Supplementary Figure S12.** Spearman correlation between NRGs expression level and IC50 level of drugs. **A** Bubble chart for CESC. **B** Bubble chart for OV. **C** Bubble chart for UCEC. **D** Bubble chart for UCS. The bubble color indicates the degree of correlation index. The bubble size indicates the *P*-value. The correlation with *P*<0.05 & |Cor|>0.3 were retained to produce the figure.

**Additional file 13: Supplementary Table 1.** The primer sequences in PCR analysis.

**Additional file 14: Supplementary Table 2.** The results of expression differences for pan-cancer analysis.

**Additional file 15: Supplementary Table 3.** The results of Univariate Cox regression analysis for pan-cancer.

## Data Availability

The RNA sequencing profiles are able to be gained from The Cancer Genome Atlas (TCGA) (https://toil.xenahubs.net). Further inquiries can be directed to the corresponding author.

## Abbreviations

GC: Gynecological cancers
CESC: Cervical squamous cell carcinoma and endocervical adenocarcinoma
OV: Ovarian serous cystadenocarcinoma
UCEC: Uterine corpus endometrial carcinoma
UCS: Uterine carcinosarcoma
NRG: Necroptosis-relate gene
GSEA: Gene Set Enrichment Analysis
MAF: Mutation Annotation Format
PCA: Principal component analysis
TIDE: Tumor immune dysfunction and exclusion
TCGA: The Cancer Genome Atlas
LASSO: Least absolute shrinkage and selection operator
RS: Risk score
OS: Overall survival
LRG: Low-risk group
HRG: High-risk group
ssGSEA: Single-sample gene set enrichment analysis
ICB: Immune checkpoint blockade
K-M: Kaplan–Meier
CNV: Copy number variation
