# Supplementary figures and images for: A comprehensive pan-cancer analysis of necroptosis molecules in four gynecologic cancers
Source: BMC Cancer. 2022 Nov 10;22:1160. doi: 10.1186/s12885-022-10166-6 (PMC9650890; doi:10.1186/s12885-022-10166-6)

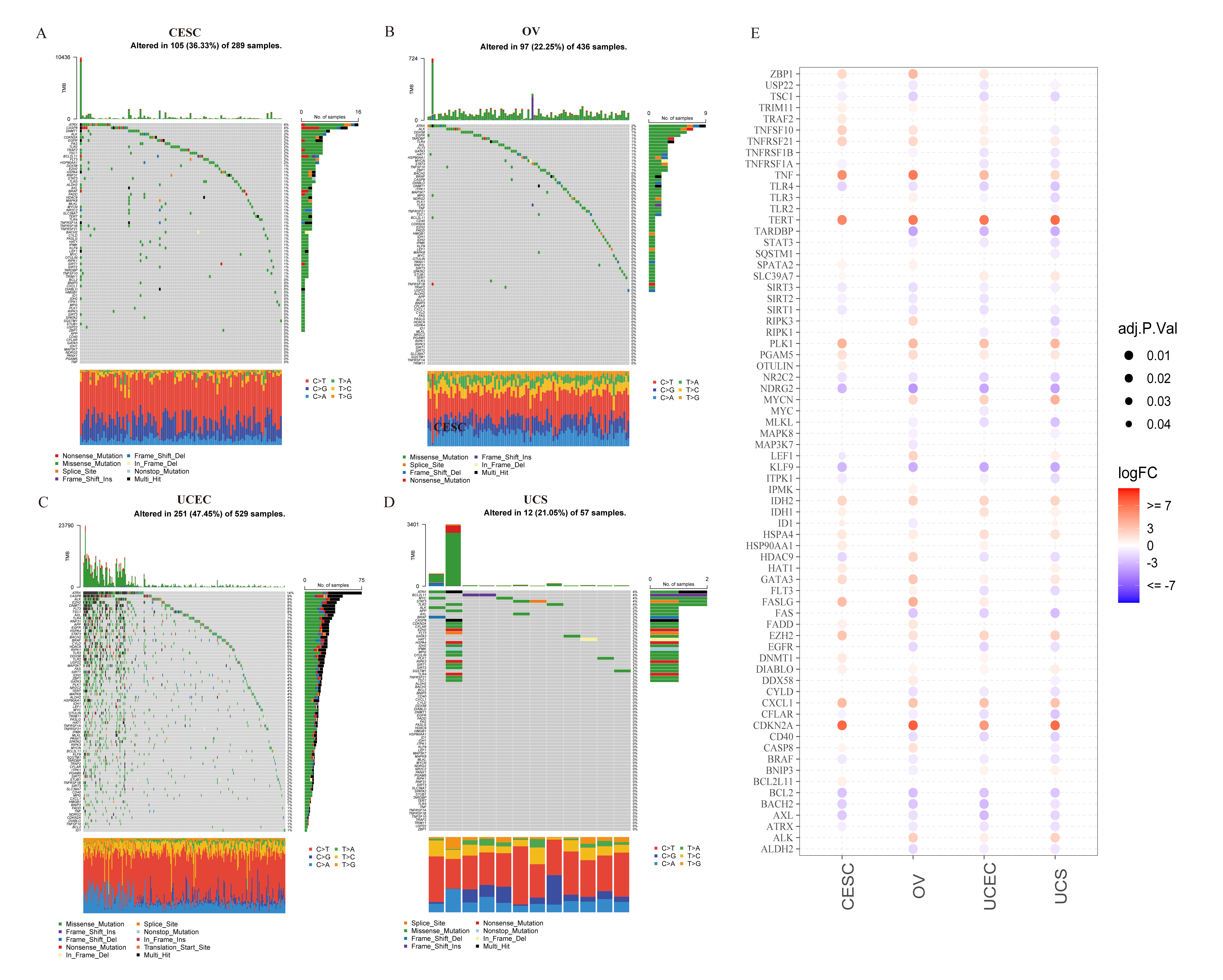

Supplement: Supplementary file 1 — Additional file 1: Supplementary Figure S1. Mutation frequency and expression variation of the 76 necroptosis-related genes (NRGs). A-D Mutation frequency of NRGs in patients with CESC (A), OV (B), UCEC (C), and UCS D. The small figure above shows the TMB, the number on the right shows the mutation frequency of each NRG, and the figure on the right shows the proportion of each vari©. E Expression levels of NRGs in four gynecological tumors. The color of the dots represents the degree of variance. Redder dots represent higher expression in cancer tissue. Bluer dots represent higher expression in normal tissue. The size of the bubbles indicates the adjusted P-value. Larger bubbles represent a lower adjusted P-value. The genes with adjusted P-value<0.05 & |logFC|>0.5 were retained to produce the figure. [file 12885_2022_10166_MOESM1_ESM.tif]

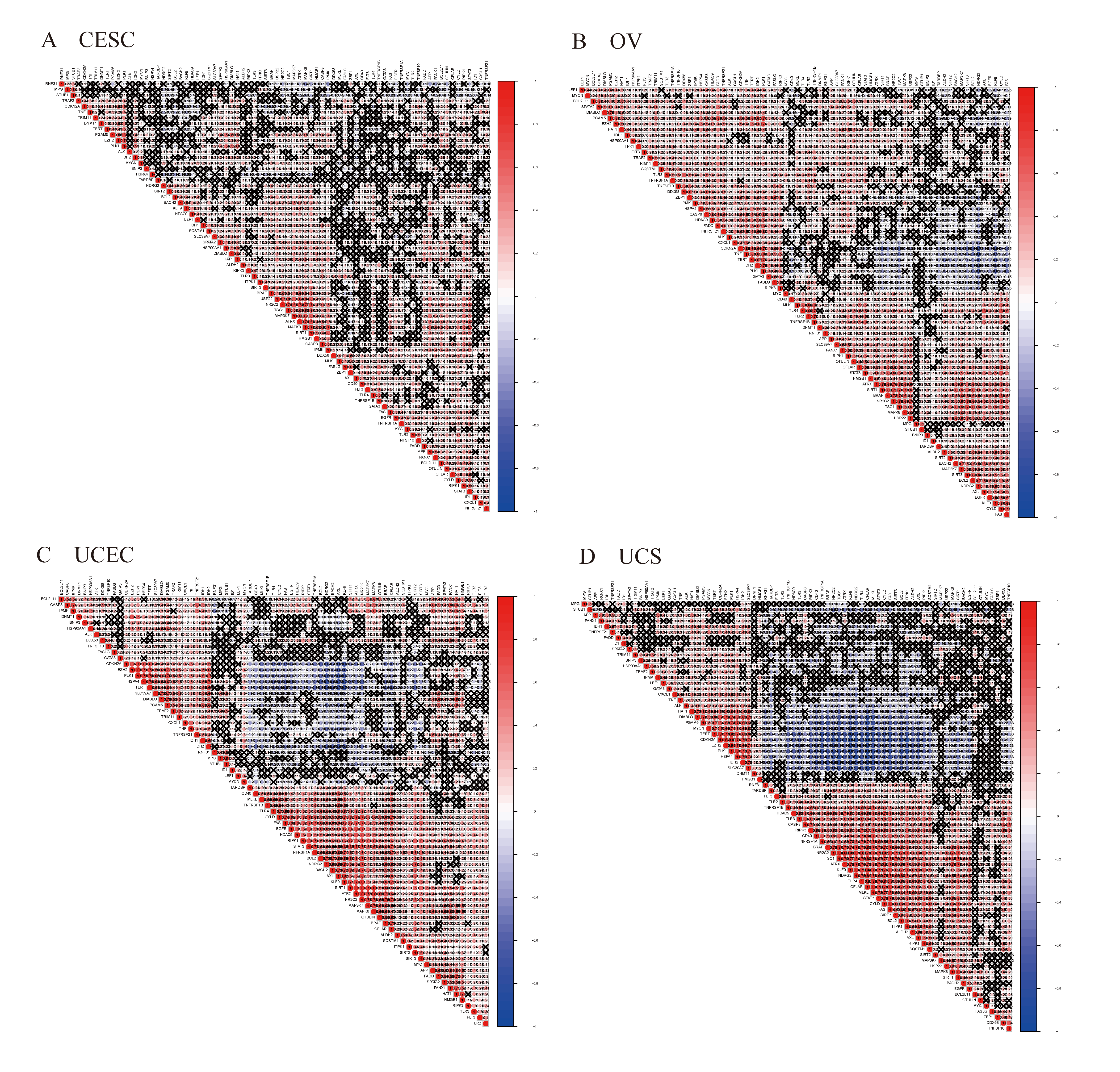

Supplement: Supplementary file 2 — Additional file 2: Supplementary Figure S2. Correlation analysis of the 76 NRGs for CESC (A), OV (B), UCEC (C), and UCS D. Red indicates a positive correlation; blue indicates a negative correlation. [file 12885_2022_10166_MOESM2_ESM.tif]

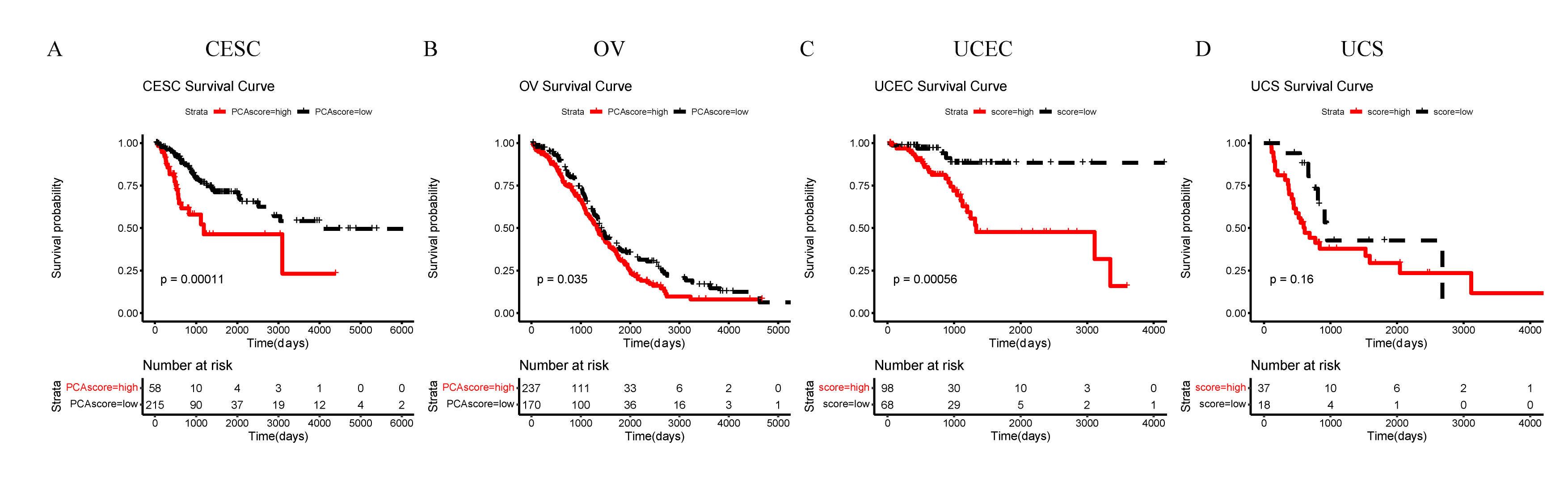

Supplement: Supplementary file 3 — Additional file 3: Supplementary Figure S3. The survival curves of necroptosis-score. A-D Kaplan–Meier curve was used to analyze the survival rate of CESC (A), OV (B), UCEC (C), and UCS (D) patients with high or low necroptosis-score. Black curves indicate low necroptosis-score and red curves indicate high necroptosis-score. [file 12885_2022_10166_MOESM3_ESM.tif]

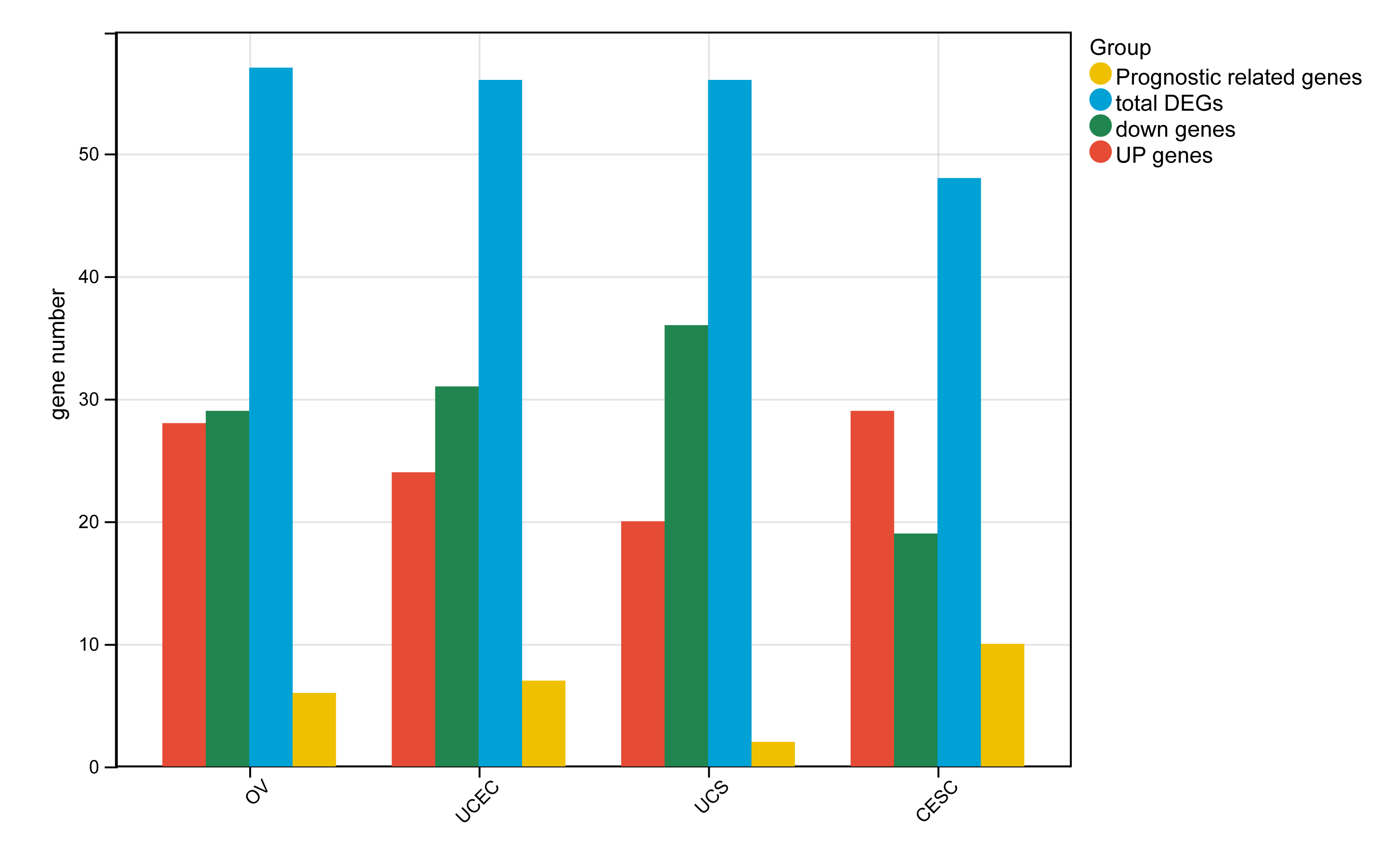

Supplement: Supplementary file 4 — Additional file 4: Supplementary Figure S4. Statistical analysis of the number of differential NRGs and prognostic NRGs of the four GCs. Red bars show up-regulated NRGs in cancer tissue. The green bars show NRGs that are downregulated in cancer tissue. The blue bars show the sum of the differential NRGs. Yellow bars indicate prognostic NRGs. [file 12885_2022_10166_MOESM4_ESM.tif]

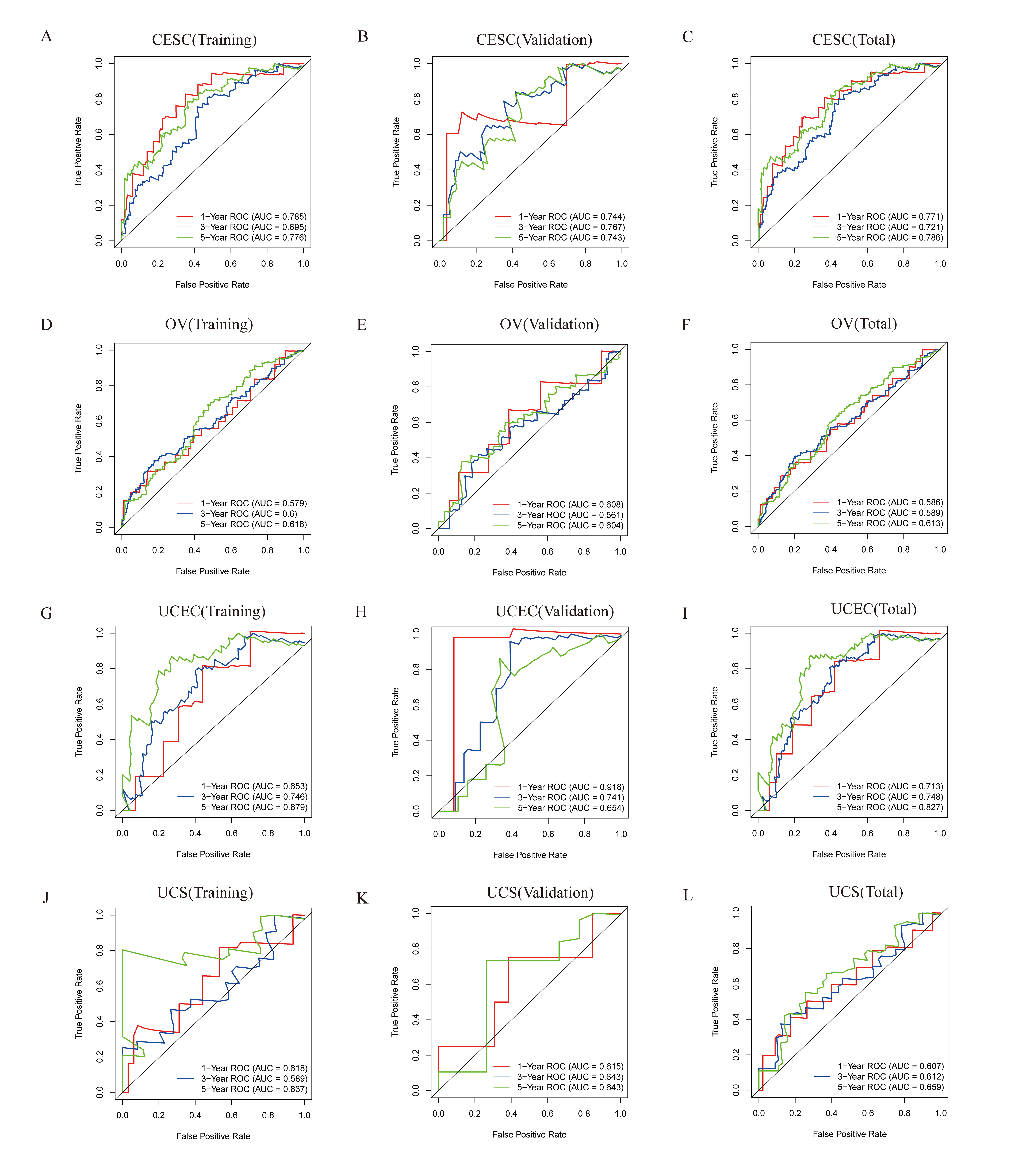

Supplement: Supplementary file 5 — Additional file 5: Supplementary Figure S5. The ROC curves for the risk model in the four GCs. A-C The ROC curves of CESC for training (A), validation (B), and total (C) sets. D-F The ROC curves of OV for training (D), valida©n (E), and total (F) sets. G-I The ROC curves of UCEC for training (G), validation (H), and total (I) sets. J-L The ROC curves of UCS for training (J), validation (K), and total (L) sets. [file 12885_2022_10166_MOESM5_ESM.tif]

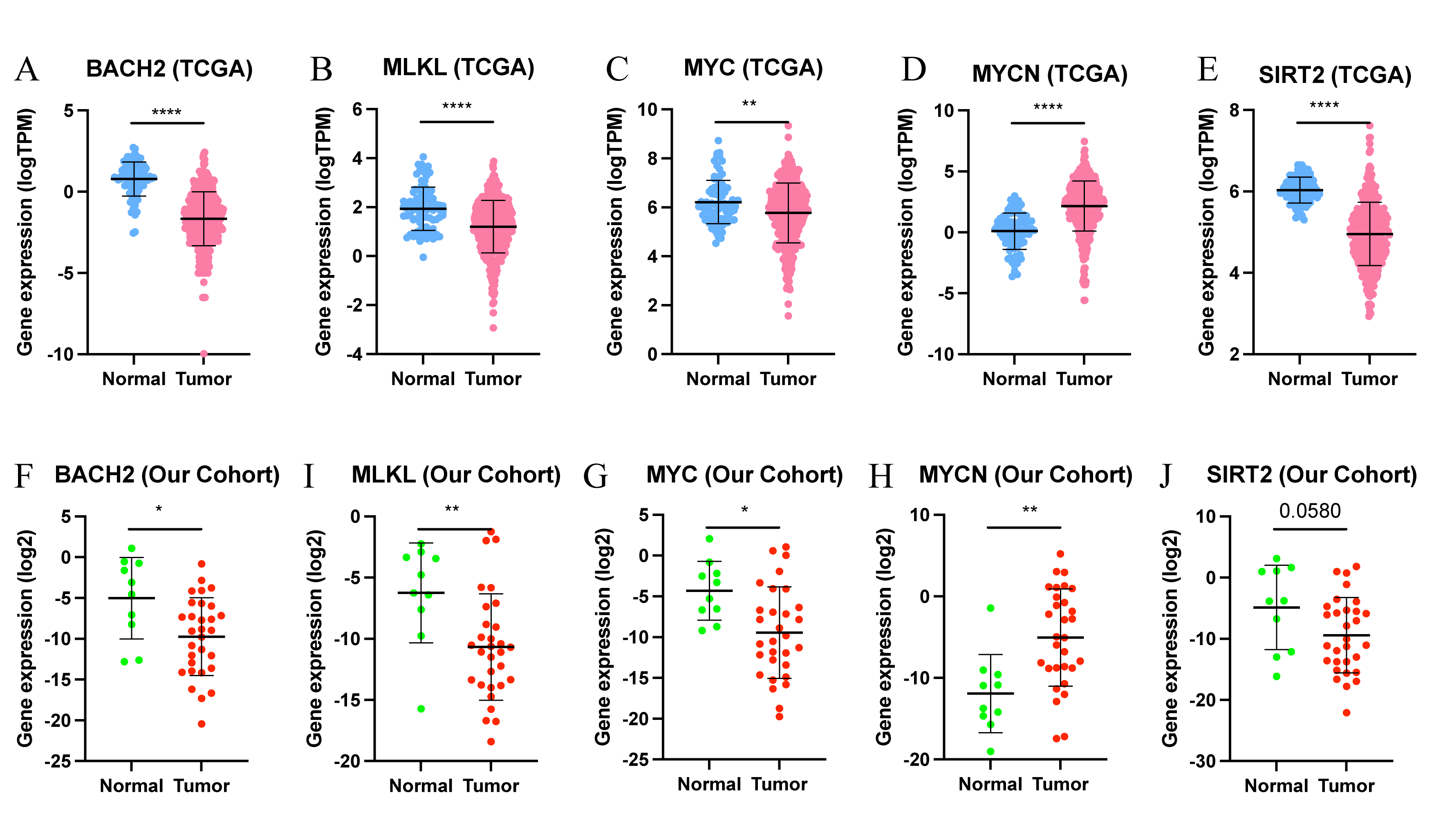

Supplement: Supplementary file 6 — Additional file 6: Supplementary Figure S6. Expression values of NRGs in prognostic signature of OV. A-D Expression values of NRGs in TCGA for BACH2 (A), MLKL (B), MYC (C), MYCN (D), and SIRT2 (E). F-J Expression values of NRGs in our cohort for BACH2 (F), MLKL (I), MYC (G), MYCN (H), and SIRT2 (J). [file 12885_2022_10166_MOESM6_ESM.tif]

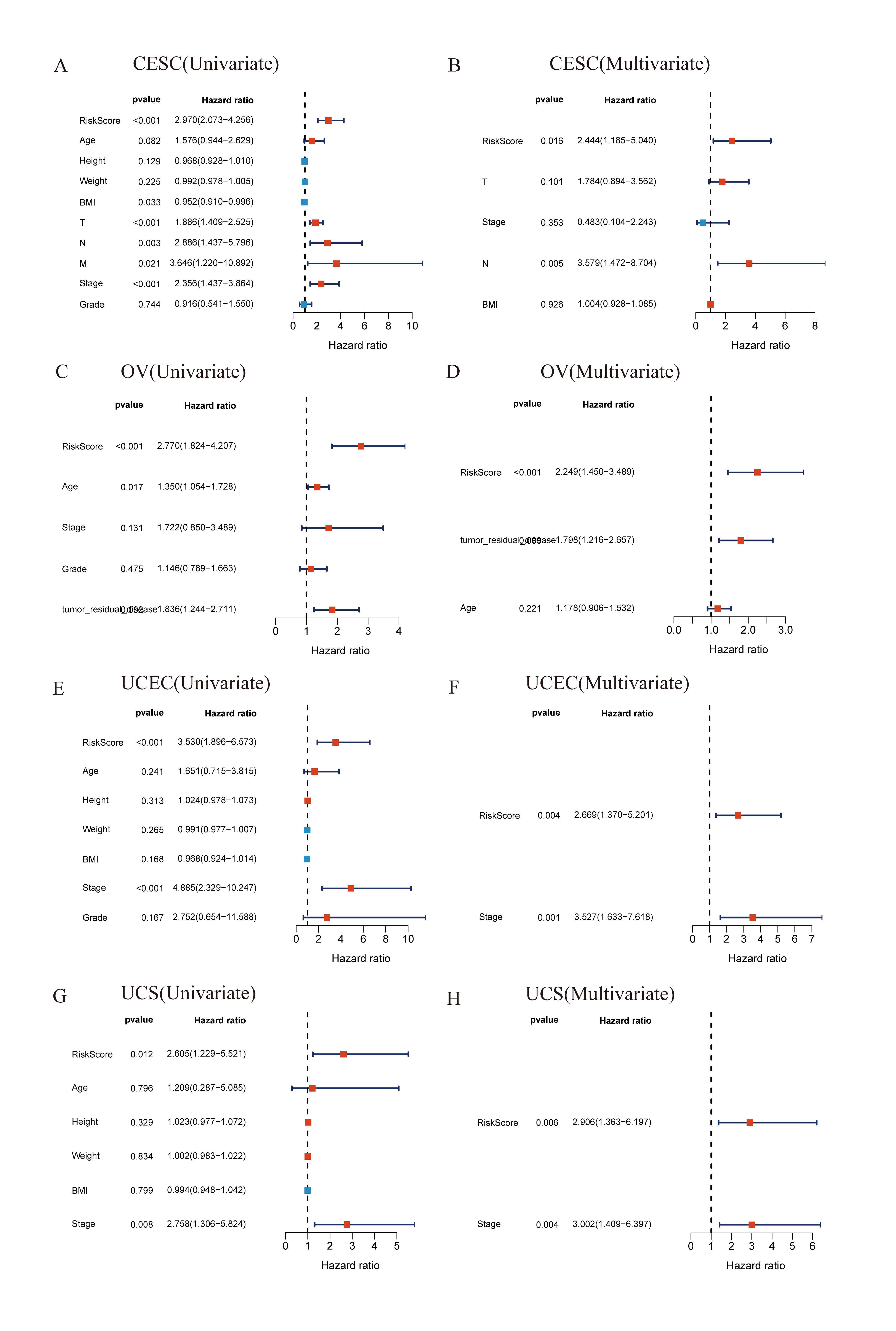

Supplement: Supplementary file 7 — Additional file 7: Supplementary Figure S7. Clinical value of risk score by independent prognostic analysis. A-H The Univariate Cox regression analysis and Multivariate Cox regression analysis for CESC (A-B), OV (C-D), UCEC (E-F), and UCS G-H. [file 12885_2022_10166_MOESM7_ESM.tif]

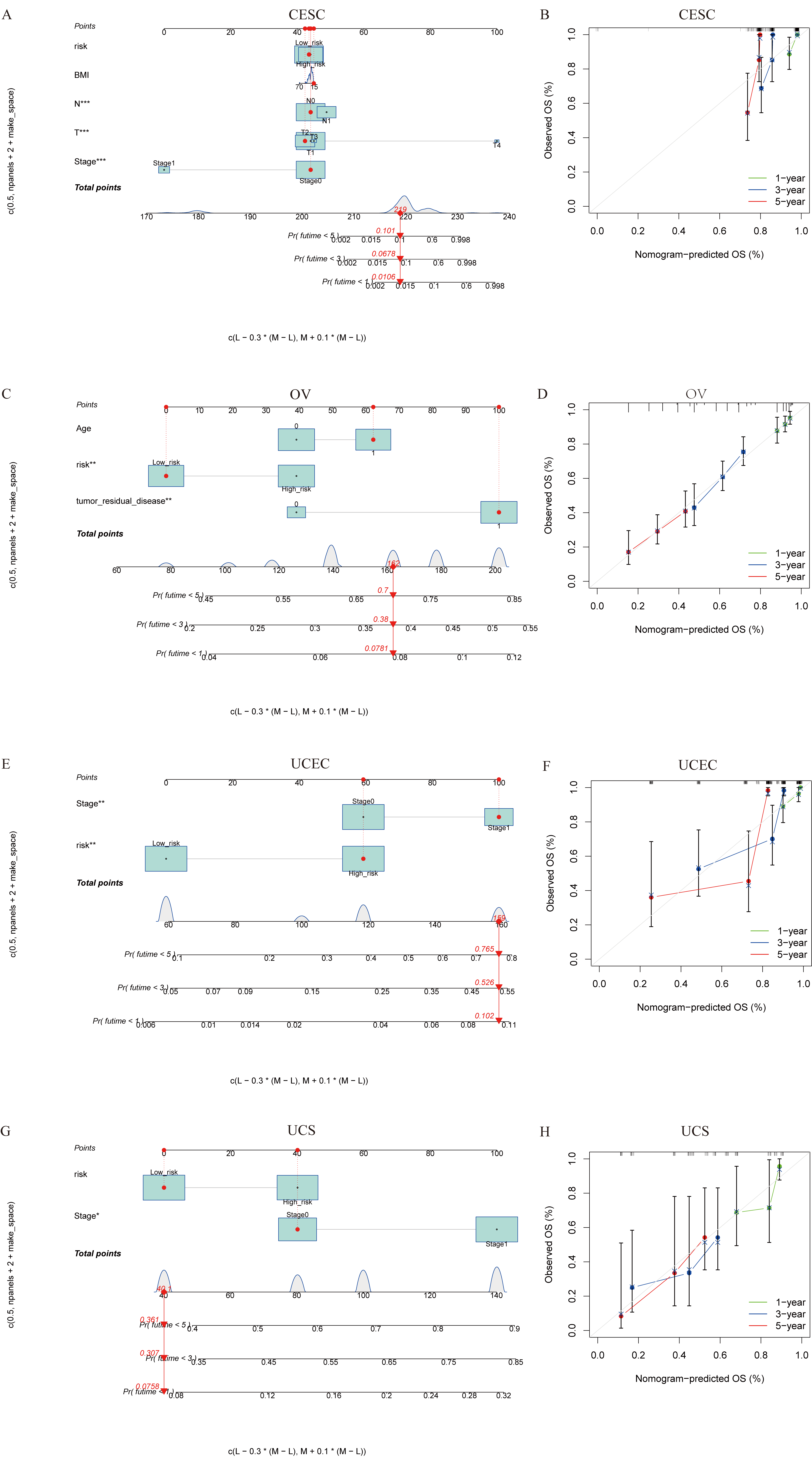

Supplement: Supplementary file 8 — Additional file 8: Supplementary Figure S8. The Nomogram model based on risk model and clinical features for GCs. A-B The Nomogram (A) and calibration curve (B) for CESC. C-D The Nomogram (C) and calibration curve (D) for OV. (E-F) The Nomo©m (E) and calibration curve (F) for UCEC. G-H The Nomogram (G) and calibration curve (H) for UCS. [file 12885_2022_10166_MOESM8_ESM.tif]

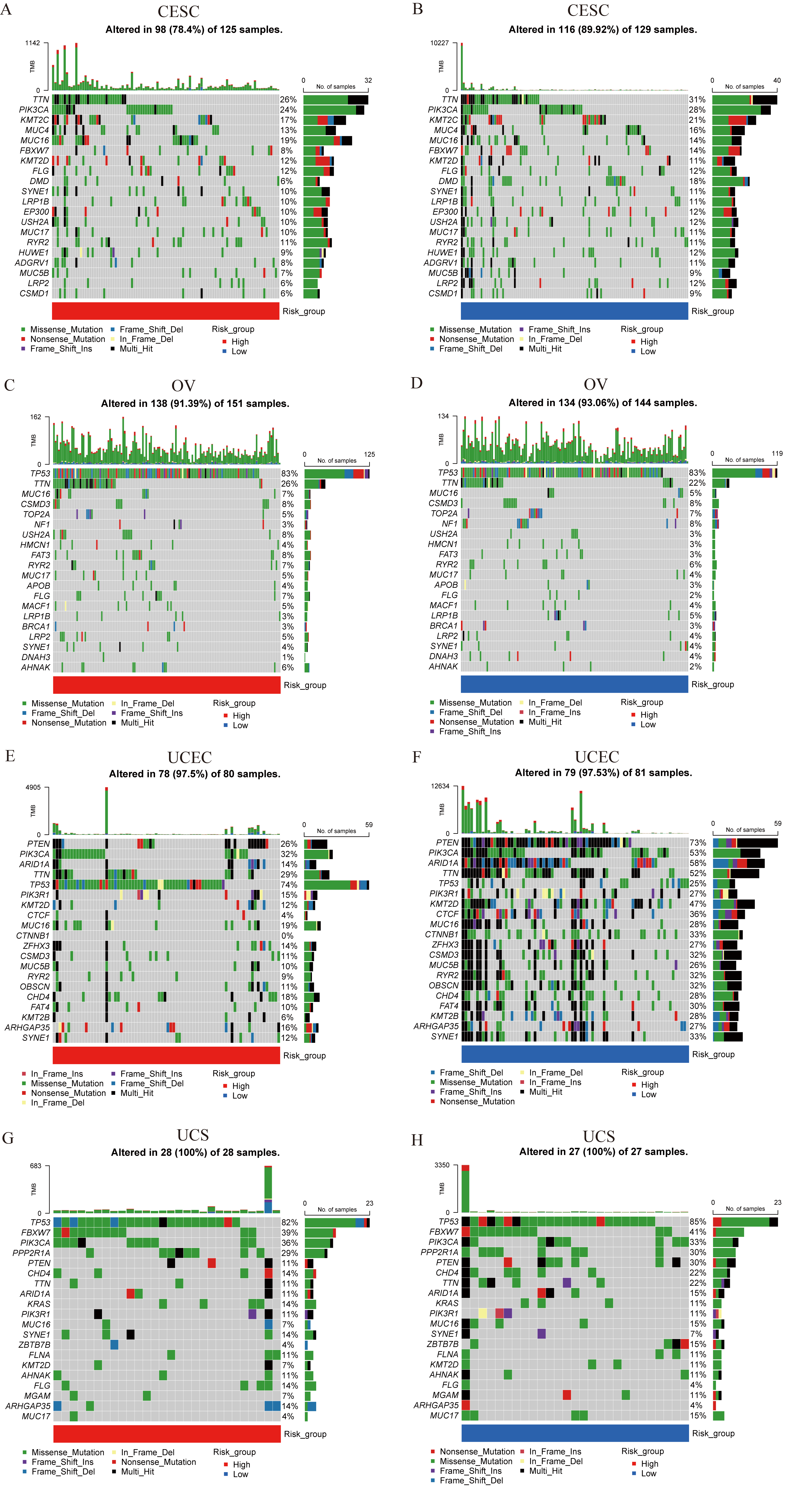

Supplement: Supplementary file 9 — Additional file 9: Supplementary Figure S9. The waterfall plot of somatic mutation features established with risk scores. A-B The waterfall plot of somatic mutation in CESC for high-risk group (A) and low-risk group B. C-D The waterfall plot of somatic mutation in OV for high-risk group (C) and low-risk group D. E-F The waterfall plot of somatic mutation in UCEC for high-risk g©p (E) and low-risk group F. G-H The waterfall plot of somatic mutation in UCS for high-risk group (G) and low-risk group H. [file 12885_2022_10166_MOESM9_ESM.tif]

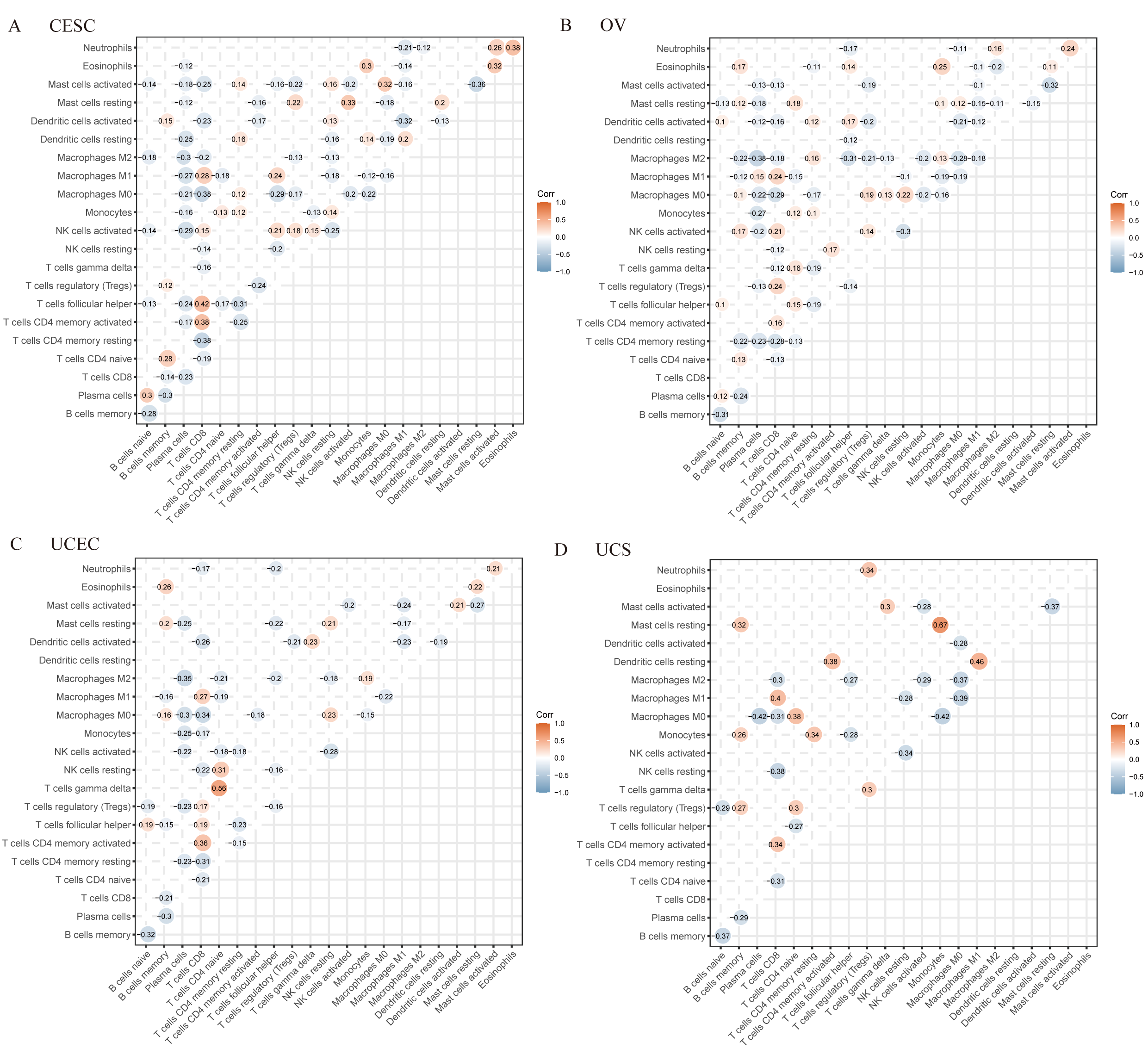

Supplement: Supplementary file 10 — Additional file 10: Supplementary Figure S10. Spearman correlation coefficient between 22 immune cells was calculated for CESC (A), OV (B), UCEC (C), and UCS D. Red bubbles indicate positive correlations and blue bubbles indicate negative correlations. The numbers in the bubbles represent the correlation coefficients. [file 12885_2022_10166_MOESM10_ESM.tif]

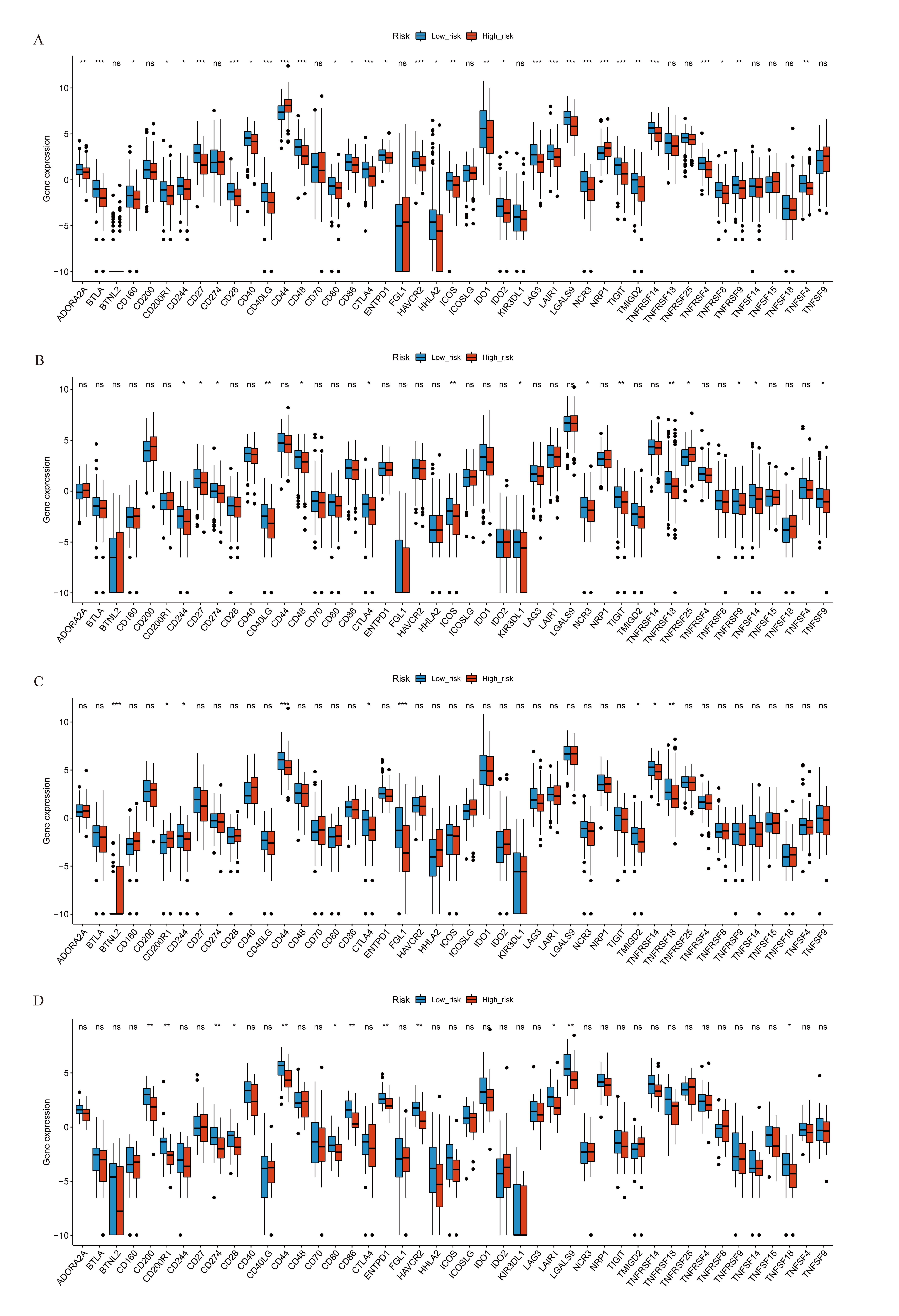

Supplement: Supplementary file 11 — Additional file 11: Supplementary Figure S11. Expression of immune checkpoints in the low and high-risk groups for CESC (A), OV (B), UCEC (C), and UCS D. ∗P<0.05; ∗∗P<0.01; ∗∗∗P<0.001; ns: not significant. The blue bars represent the low-risk group and the red bars represent the high-risk group. [file 12885_2022_10166_MOESM11_ESM.tif]

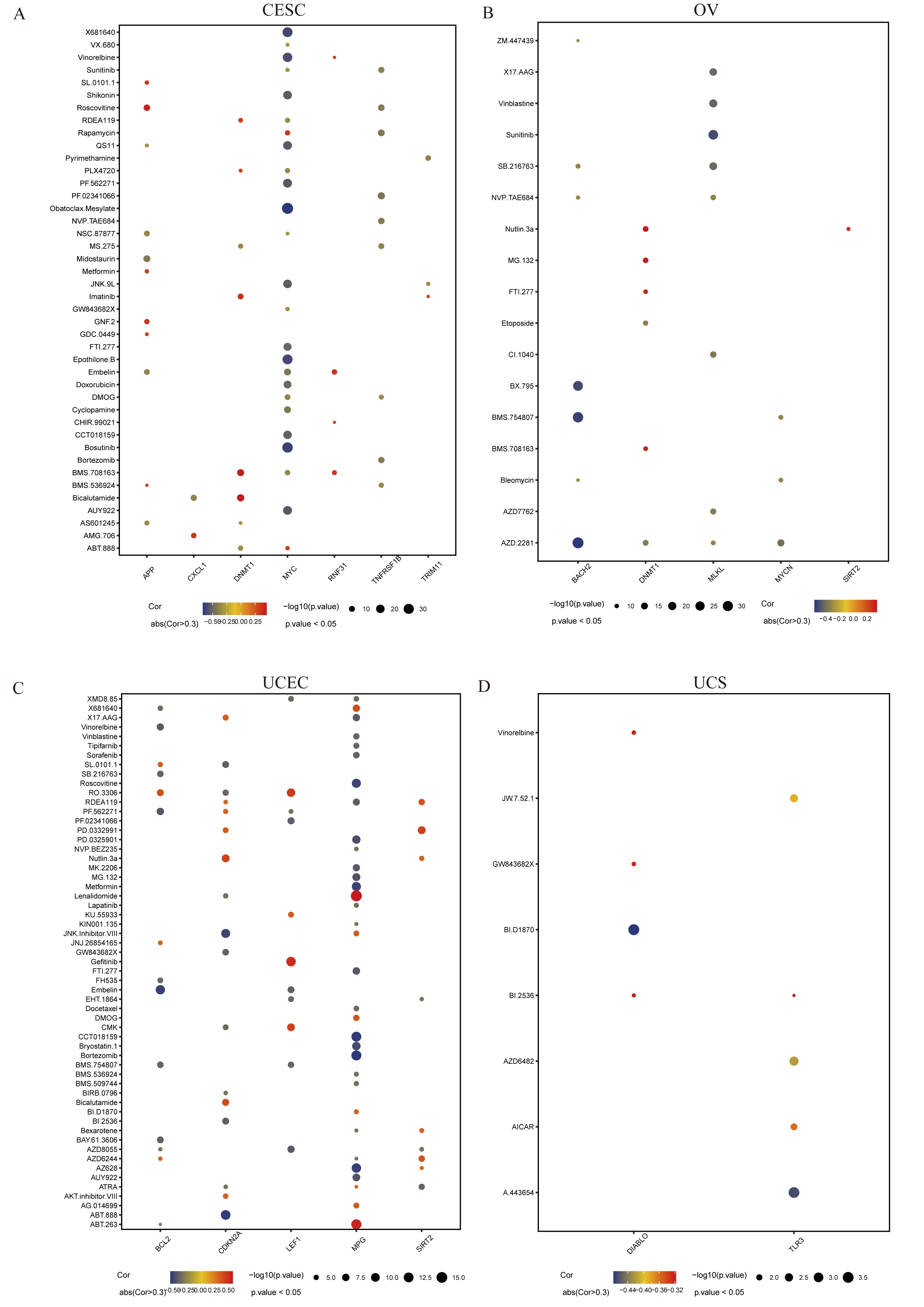

Supplement: Supplementary file 12 — Additional file12: Supplementary Figure S12. Spearman correlation between NRGs expression level and IC50 level of drugs. A Bubble chart for CESC. B Bubble chart for OV. C Bubble chart for UCEC. D Bubble chart for UCS. The bubble color indicates the degree of correlation index. The bubble size indicates the P-value. The correlation with P<0.05 & |Cor|>0.3 were retained to produce the figure. [file 12885_2022_10166_MOESM12_ESM.tif]
